# Polarization and Dipole Moment Effects on Sigma‐Hole Potential in Tin(IV)‐Porphyrins

**DOI:** 10.1002/chem.202502099

**Published:** 2025-07-22

**Authors:** Rafia Siddiqui, Raphael F. Ligorio, Hatem M. Titi, Sushil Kumar Pandey, Anna Krawczuk, Ranjan Patra

**Affiliations:** ^1^ Amity Institute of Click Chemistry Research and Studies Amity University Noida Uttar Pradesh 201303 India; ^2^ Institute of Inorganic Chemistry University of Göttingen Tammannstrasse 4 D‐37077 Göttingen Germany; ^3^ Department of Chemistry McGill University 801 Sherbrooke St. West Montreal QCH3A0B8 Canada; ^4^ Aragen Life Sciences Ltd. Mallapur Secunderabad Telangana 500076 India

**Keywords:** dipole moment, halogen bonding, metalloporphyrins, polarization, supramolecular chemistry

## Abstract

This study investigates how electron‐withdrawing substitution, molecular polarization, and dipole moment influence the σ‐hole potential in six‐coordinate metalloporphyrins. To evaluate halogen bonding tendencies, we synthesized a series of five Sn(IV)‐5,10,15,20‐meso‐tetrakis(4‐iodophenyl)porphyrin complexes with various fluorinated phenolate axial ligands. Single‐crystal X‐ray diffraction analysis revealed distinct halogen‐bonded supramolecular motifs, which vary depending on the degree of fluorination at the axial ligands. Our findings highlight the critical role of ligand‐induced polarization and dipole moment variations in modulating the σ‐hole characteristics of the equatorial iodine atoms. Computational modelling showed that increased fluorine substitution reduces both the atomic dipole moments of fluorine and the polarizability of the central tin ion. However, despite these changes, the axial fluorination has a negligible effect on the σ‐hole potential at the iodine atoms. This limited influence is attributed to the orthogonal orientation between the porphyrin core and the peripheral phenyl rings, which suppresses resonance interactions. Overall, this work emphasizes the importance of understanding electronic effects at the molecular level, particularly in the design and formation of halogen‐bonded supramolecular architectures.

## Graphical abstract

Through this work with experimental and computational studies we have systematically investigated the role of interplay of electronic effects such as polarization, dipole moment and resonance on the sigma‐hole potential in macrocyclic system like, metalloporphyrin system. The behavior of the sigma‐hole potential of equatorial iodine atom of metalloporphyrin has been studied upon changing substituted ligands at axial position in metalloporphyrin.

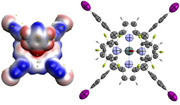

## 1 Introduction

Porphyrins represent a unique class of heterocyclic tetrapyrrolic organic molecules that are ubiquitous in nature. They are of fundamental importance to help sustain life on this planet through storing and transporting oxygen (as observed in hemoglobin), enabling photosynthesis (through chlorophyll), and serving as constituents of numerous enzymes and vitamins.^[^ ^1^, ^2^, ^3^, ^4^, ^5^ ^]^ These aromatic macrocycles are remarkable building blocks for self‐assembly due to their intimate packing, structural characteristics and molecular recognition properties. They can be easily diversified by incorporating different functional groups on the periphery and/or the axial positions of the porphyrin skeleton. These functionalized porphyrins result in both covalent and non‐covalent porphyrin assemblies, which possess a major scope in various research fields.^[^ ^5^, ^6^, ^7^, ^8^, ^9^, ^10^ ^]^ A wide variety of novel porphyrin‐based supramolecular architectures, assembled primarily through intermolecular coordination and hydrogen‐bonding (HB) interactions, have been reported in recent years.^[^ ^11^, ^12^, ^13^, ^14^ ^]^ Among those, HB and π−stacking interactions have been considered the leading players in this field for decades. However, more recently, halogen bonding (XB) interactions were recognized as a front‐runner of non‐covalent interactions that can be used as a novel tool for controlled supramolecular self‐assembly.^[^ ^15^, ^16^, ^17^, ^18^, ^19^, ^20^ ^]^

Unlike traditional HBs, XBs involve halogen atoms (X) with much higher polarization, leading to more robust and directional interactions. This strength and directionality enable precise control over the arrangement of molecules tailoring various supramolecular architectures. Nonetheless, metalloporphyrin assemblies with the aid of XBs have received minimum attention. In their pioneering work, Goldberg's group has demonstrated the effectiveness of directional I···I and Br···Br interactions during self‐assembly crystallization.^[^ ^21^, ^22^, ^23^ ^]^

Some of us have explored the self‐assembly with distinguished metalloporphyrin systems via halogen bonding and also investigated the positive potential on their metal centres of metalloporprhyrins.^[^ ^20^, ^24^, ^25^, ^26^, ^27^, ^28^, ^29^ ^]^ Our recent work with six‐coordinated Sn(IV)‐5,10,15,20‐*meso*‐tetrakis‐(4‐iodophenyl) porphyrin [Sn(L)_2_‐TIPP] porphyrins by varying the axial ligands position led to different ladder‐like supramolecular synthons, 1D or 2D halogen bonded networks, without activating the substituted Br/I *meso*‐positions in Sn(IV)‐porphyrins.^[^ ^26^ ^]^ It is well known that crystal structures of porphyrins are mainly influenced by dispersion forces between aromatic fragments and molecular shape, while HB and XB interactions play a smaller attribution to the cohesive energy of the crystal packing.^[^ ^30^ ^]^

**Scheme 1:**
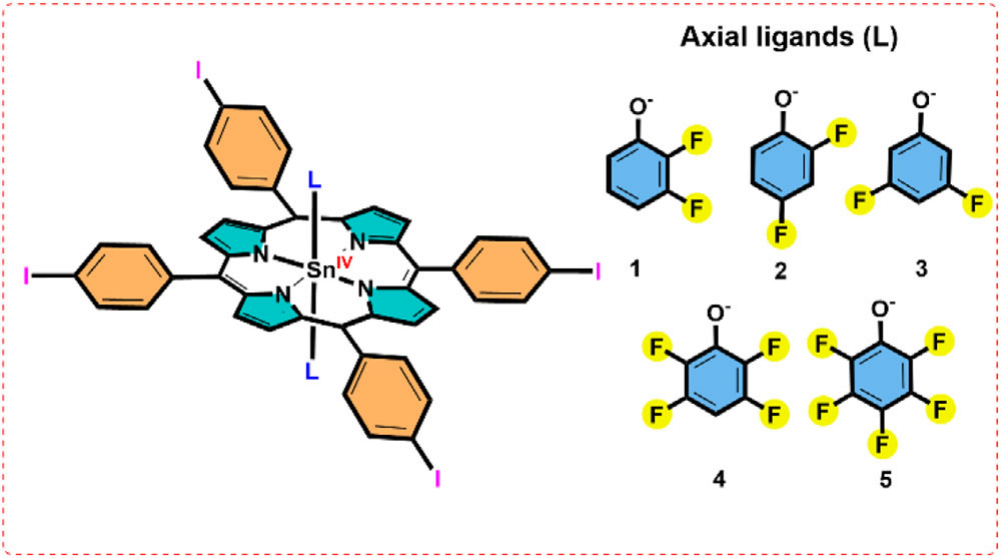
Schematic illustration of the Sn(IV)‐5,10,15,20‐*meso*‐tetrakis(4‐iodophenyl)porphyrin scaffolds obtained from single crystal X‐ray diffraction data. Axial ligands (L): 2,3‐difluoro phenol (**1**), 2,4‐difluoro phenol (**2**), 3,5‐difluoro phenol (**3**), 2,3,5,6‐tetrafluoro phenol (**4**), 2,3,4,5,6‐pentafluoro phenol (**5**). Labelling scheme of each complex is given in SI file in Figures S1–S5.

After delivering classified non‐covalent interactions in metalloporphyrin assembly our group is interested to classify the electronic effects in metalloporphyrin systems. In supramolecular chemistry, the electronic effects like polarization, electronegativity, and dipole moment are well understood but are majorly being explored in small molecules. Though it is much important to understand the influence of electronic effect in larger macromolecules like porphyrins, yet it is highly challenging. This is because dispersion forces and other intermolecular forces mainly control interactions in these assemblies. Therefore, to explore the above‐mentioned electronic effects at macrocyclic level, we have designed Sn(TIPP)^2+^(L^−^)_2_ scaffolds with successive addition of a fluorine atom at the axial phenolate (Scheme 1).

In all the complexes, the structural stability arises from a combination of various weak interactions, such as C‐H**···**X, C‐H**···**π and π**···**π, to collectively surpass the influence of X**···**X interactions and dominate the crystal packing. The electronic effect of axial ligands is scrutinized through a DFT study followed by QTAIM analysis. To the best of our knowledge, this is the first time the nature of halogen bonding in metalloporphyrins has been systematically addressed, considering the interplay of polarizability, dipole moment, and electron‐withdrawing substitution effects to understand the self‐assembly processes in porphyrin and metalloporphyrin systems.

## 2 Results and Discussion

Our main objective was to construct crystalline metalloporphyrin architectures primarily stabilized by halogen bonding. To this end, we designed tin–porphyrin complexes that incorporate both halogen bond donor sites (i.e., polarizable iodine substituents) and acceptor functionalities (e.g., X, N, or O atoms bearing lone pairs). Previous studies by some of us, have demonstrated that different types of halogen bonding (XB), particularly Type I and Type II, can play a decisive role in shaping the supramolecular architecture of five‐ and six‐coordinate metalloporphyrins through rational axial ligand design.^[^ ^24^, ^25^, ^26^, ^27^, ^28^, ^29^ ^]^ In general, the intermolecular approach in homo‐ and hetero‐halogen interactions follows a C–X⋯X–C motif, involving complementary δ⁺ and δ⁻ regions on the halogen atoms. Depending on the mutual orientation of the interacting partners, two geometries are possible: head‐on and side‐on, giving rise to Type I (symmetrical) and Type II (bent) interactions, respectively.^[^ ^26^ ^]^

### 2.1 Crystal Structure Analysis

Analysis of the crystal structures of the five Sn(IV)‐porphyrin complexes reveals that Type II interactions are predominant. One complex clearly exhibits a Type I interaction, while another displays features of both geometries, suggesting a hybrid motif (Table 1). These structural variations highlight the influence of axial ligand substitution on halogen bond topology. In light of these findings, the present study further explores how electronic effects, such as polarization and dipole moment, influence the potential and directionality of iodine sigma‐holes. This is investigated through an integrated approach combining crystallographic, electrochemical, and theoretical analyses.

**Table 1:** Intermolecular halogen bond interactions in complexes **1**–**5**.

Complex	D(X···X΄/O/π)	θ_1_(C − X···X΄)/ θ_2_(X΄···X − C)	D(X··· Y) / Å	Type of interaction
**1**	I1···I2^i^	174.34/103.7	3.914(1)	Type II
	I2···O1^ii^	167.1/112.1	3.023(6)	Type II
**2**	I1···I2^iii^	170.8/112.3	3.952(7)	Type II
	I1···F3^iv^	116.8/103.7	3.444(7)	Quasi Type
	I2···O1^v^	165.7/110.6	3.068(3)	Type II
	I2···C1^v^	100.9/90.6	3.651(4)	Type I
**3**	I1···I2^vi^	172.8/95.9	3.759(6)	Type II
	I1···F1^vii^	155.9/115.4	3.285(6)	Type II
**4**	I2···F2^viii^	164.2/137.2	3.323(5)	Type II
**5**	I1···I2^ix^	173.6/96.5	3.811(6)	Type II
	I1···C19^x^	162.9/94.8	3.459(5)	Type II
	I1···C20^x^	157.7/99.1	3.589(5)	Type II

Symmetry codes: (i) x + 1/2,‐y + 1/2 + 1,z + 1/2; (ii) ‐x + 1/2 + 1,y + 1/2,‐z + 1/2 + 1; (iii) x‐1/2,‐y + 1/2 + 1,z‐1/2; (iv) x‐1/2,y + 1/2,+z; (v) x,‐y + 1,z + 1/2; (vi) ‐x + 1,‐y + 2,‐z + 2; (vii) ‐x,‐y + 2,‐z + 1; (viii) x + 1,+y‐1,z; (ix) x‐1/2, y + 3/2, z; (x) ‐x + 1,+y‐1,‐z + 3/2.

**Figure 1:**
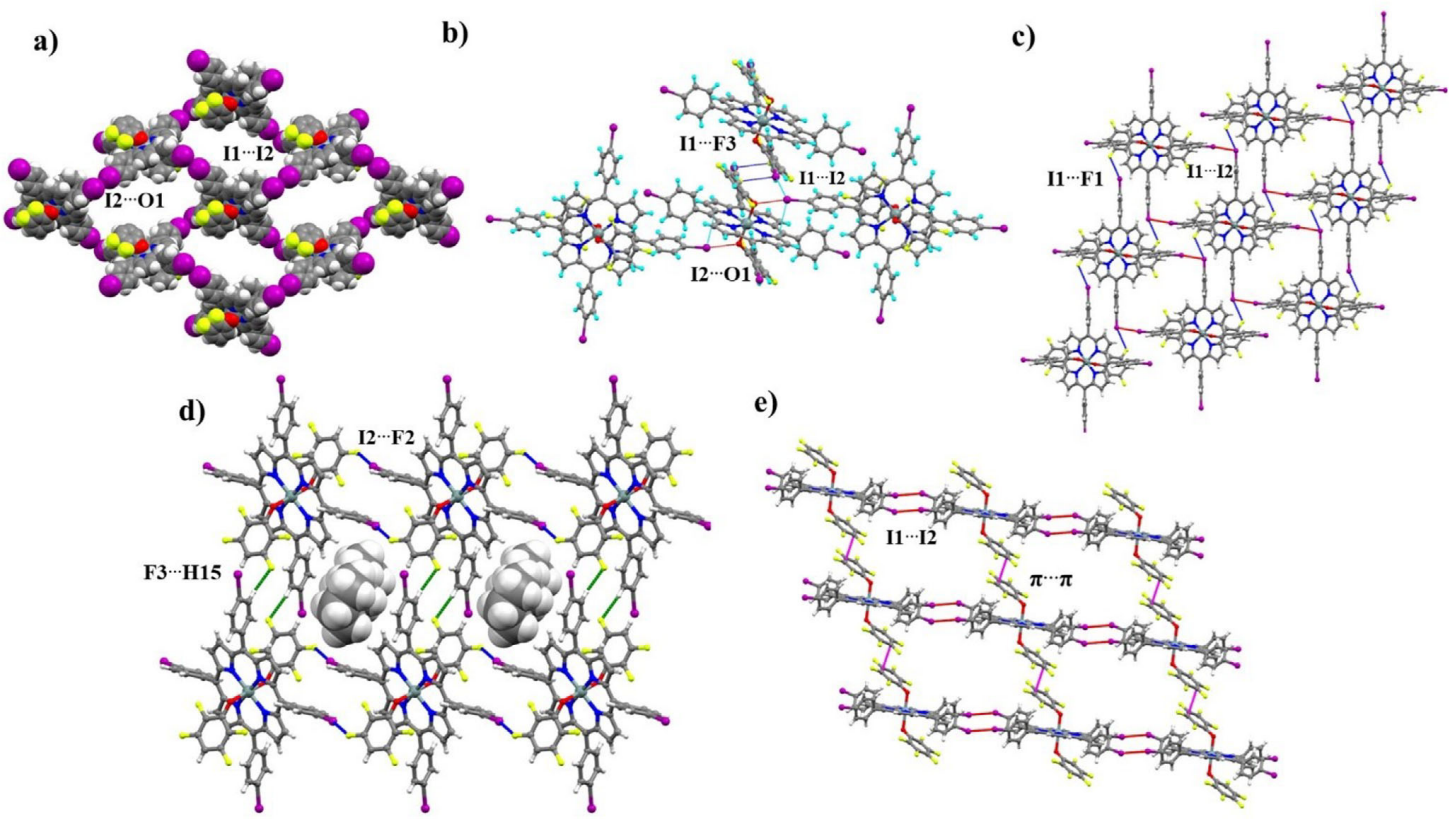
a) Supramolecular 2D halogen‐bonded network arrays are used in the space‐fill model of complex **1**. b) Bifurcated XB between F‐atoms (green colour), and I‐atoms (purple colour), I1···F3 while we also observed a trifurcation of XB interactions involving I2 atom with phenolate oxygen O1 (I2···O1) in complex **2**. c) Cooperative XB interactions involving I1···F1 (blue dotted lines) and I1···I2 (red dotted lines) interactions in complex **3** makes 2D stair‐like supramolecular networks. d) Cooperative inter‐halogen interactions involving I2···F2 (blue dotted lines) and hydrogen bond interactions F···H (green dotted lines) interactions in complex **4** makes 2D supramolecular networks. Solvent cyclohexane is trapped in the cavity and generated through cooperative halogen and hydrogen bonds. e) XB interaction in complex **5**, I1···I2 type‐II halogen bond interactions (red dotted line) in complex **5** and cooperative π···π stacking between the pentafluoro‐phenyl group (pink dotted line) leads to 2D supramolecular network. Disorder solvents were omitted for clarity.

Complex **1** crystallizes in a monoclinic space group, *C*2/*c*, from a mixture of CHCl_3_:cyclohexane (1:1, v/v) by slow evaporation at room temperature. In this case, the axial positions are substituted with 2,3‐difluorophenolate moiety, whereas the *meso*‐positions contain two different iodine environments, I1 and I2. In which I1 substituent interacts with the equatorial I2 of a neighbouring porphyrin to form Type II XB with a distance of 3.914 (1) Å, shorter by 0.046 Å than the sum of their corresponding van der Waals (vdW) radii.^[^ ^31^, ^32^, ^33^ ^]^ The second equatorial iodine atom, I2, is instead involved in an interaction with an adjacent phenolate oxygen atom via I···O contact with a distance of 3.023(7) Å, which is slightly longer than the reported contact of I···O type by Goldberg, 2.947 Å, (CSD Ref. Code LUGMOR).^[^ ^34^ ^]^ Noteworthy, the F‐atoms play no role in the self‐assembly of complex **1**. The I^…^I and I···O interactions between iodine species lead to a brick‐like 2D fused L‐node XB 1‐point motif (Figure 1a).

Moving one of the F‐atom positions from *meta* to *para* by using 2,4‐difluoro phenolate as an axial ligand in complex **2**, where *meta* positions display a disorder with half occupancy on each F‐atom. This complex also crystallized in a monoclinic space group, *C*2/*c*, from a mixture of dimethylformamide (DMF) and chloroform, in which the asymmetric unit consists of a half porphyrin molecule, one chloroform and disorder DMF solvates (see Figure S2). Similar to **1**, complex **2** is also involved in equatorial iodine atoms of metalloporphyrins are involved in I···I and I···O interaction, in addition to weak interactions such as I···F and localized I···π interactions.^[^ ^35^, ^36^, ^37^ ^]^ In this case, I1 substituent is involved in bifurcation interactions with I2 atom (Type II) and F3 (Quasi Type) of the adjacent porphyrin with distances of 3.952(7) and 3.444(5) Å, respectively (Figure 1b and ESI). On the other hand, atom I2 is engaged in trifurcation interactions. In addition to I···I, the I2 atom is forming a weaker interaction with the O‐atom of the phenolate with the distance of 3.068(3) Å. The Friščić group found that 5‐member rings rarely form I···π interactions, contributing only 2% of total halogen bonds in the CSD (the threshold was set to be 3.68 Å for such interactions to occur), which made I···Cα_π_ in **2** attractive *semi‐localized* interaction and slightly shorter than the threshold, 3.650(5) Å.^[^ ^38^ ^]^ In this case, the overall packing is also considered as 2D fused L‐node motif.^[^ ^39^ ^]^

Switching to meta‐substitution with F‐atoms using 3,5‐difluorophenolate resulted in a different supramolecular architecture in complex **3**. This complex crystallizes in a triclinic space group, *P*‐1, with half a molecule in the asymmetric unit (see Figure S3). One of the iodine atoms exhibits bifurcated behaviour, but unlike complex 2, both homo‐halogen (I···I, 3.759(6) Å) and hetero‐halogen (I···F, 3.285(6) Å) interactions are observed simultaneously and possess Type II based contacts. These cooperative homo‐ and hetero‐halogen interactions create a 2D 1‐point 4‐ring and L‐node motif (Figure 1c).^[^ ^39^ ^]^

Complex **4** features a tetrafluorophenolate group at the axial position and crystallizes with disordered cyclohexane solvent molecules in the triclinic space group P‐1 (see Figure S4). This derivative forms a unique halogen bond network compared to earlier complexes. Here, iodine (I2) only engages in a hetero‐halogen bond with fluorine (F2) at a distance of 3.323(6) Å, while iodine (I1) forms a hydrogen bond with a cyclohexane solvent molecule. Among the four fluorine atoms, F1 does not participate in any interactions, F3 forms a hydrogen bond with *meso*‐phenyl hydrogen (C15‐H15⋯F3, d = 2.501(6)Å, ∡C–H⋯F = 173.2°), and F4 engages in a halogen···π interaction with pyrrolic C_π_ in semi‐localized fashion (3.061(5) Å). These cooperative hydrogen and halogen bonds create a cage‐like structure that encloses the cyclohexane solvent molecules (see Figure 1d), similar to those observed in Sn(tetrafluorobenzoate)_2_tetraiodophenylporphyrin complexes.^[^ ^26^ ^]^ The structural halogen bonded network is recognized as 1D with a 2‐point motif.^[^ ^39^ ^]^

Complex **5**, featuring a perfluorinated phenolate group at the axial position, crystallizes in the monoclinic *C*2/*c* space group from a chloroform‐diethyl ether solution (1:1 v/v) (see Figure S5). Interestingly, despite the five F‐atoms in the axial position, none of those atoms are involved in any type of XB interaction. However, the equatorial iodine atoms are forming Type II I···I interactions with a distance of 3.811(6) Å (see Table 1). Additionally, the adjacent porphyrin molecules participate in a π‐π stacking interaction at a distance of 3.325(2) Å, between fluorinated phenyl groups. These cooperative halogen bonds and π‐π stacking interactions create a 3D fused L‐node 1‐point motif supramolecular network, as shown in Figure 1e.^[^ ^39^ ^]^

The detailed structural and packing analyses of complexes **1**–**5** clearly reveal the formation of different halogen‐bonded supramolecular synthons depending on the degree of fluorination at the axial phenolate group. Most interestingly, halogen bond interactions with other non‐covalent interactions like hydrogen bonds and π···π stacking play a significant role in the thermodynamic stability of the studied crystal. Those interactions lead to the formation of different supramolecular synthons observed in complexes **1–5**. Hirshfeld surfaces analysis using Crystal Explorer^[^ ^40^, ^41^, ^42^ ^]^ of all the structures revealed that the I‐atom attributes account for 10–12% of the total intermolecular interactions in the crystal packing (see Figures S16‐S20). The results reveal that the F‐atoms at the axial position do not influence the I‐atoms in the *meso*‐positions.

### 2.2 Electrochemical Study

**Figure 2:**
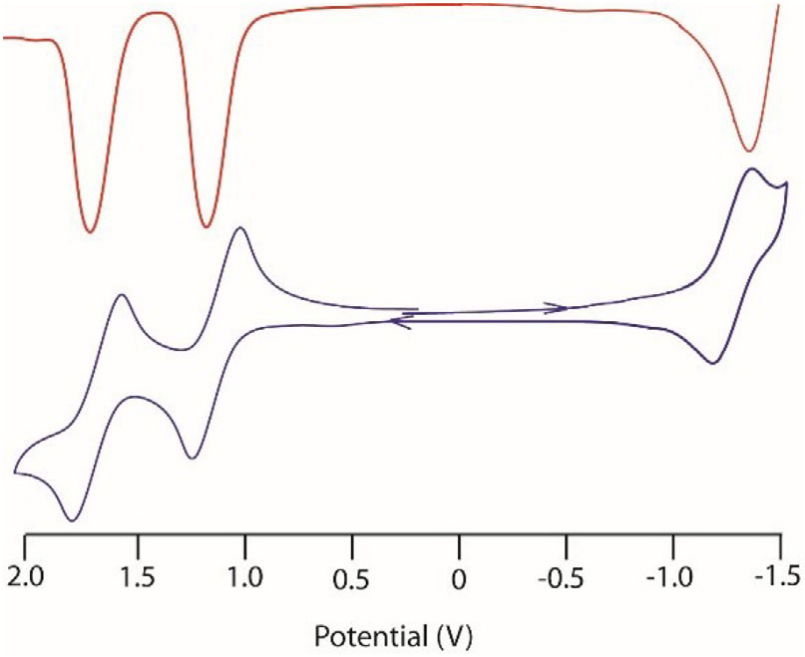
Cyclic voltammogram (blue) and differential pulse voltammetry (DPV) of complex **4** (in red) in CH_2_Cl_2_ (scan rate 100 mV s − 1) with 0.1 M tetra(n‐butyl) ammonium perchlorate as supporting electrolyte at 25 °C. The reference electrode is Ag/AgCl.

To gain further insight into the electronic influence of axial ligand fluorination on Sn(IV) porphyrins, we performed cyclic voltammetry (CV) measurements for all complexes at 25 °C in CH₂Cl₂ with 0.1 M tetrabutylammonium perchlorate (TBAP) as supporting electrolyte (Figure 2 and Figure S21). This analysis provides direct information about the redox behavior and electronic structure of the porphyrin macrocycle in response to variations in axial substitution.

Complex **4** exhibits two quasi‐reversible oxidation processes at 1.08 and 1.52 V, assigned to the formation of mono‐ and dicationic porphyrin species, as well as an irreversible reduction at − 1.52 V corresponding to the formation of a radical anion. Similar electrochemical profiles were observed for the remaining complexes, with detailed data provided in Table S3.

Importantly, systematic trends emerge upon increasing the degree of fluorination at the axial positions. Specifically, the first oxidation potentials shift anodically and the reduction potentials shift cathodically across the series, indicating progressive stabilization of both frontier orbitals. These shifts, although moderate in magnitude, reflect the cumulative electron‐withdrawing nature of the fluorinated ligands. This supports our conclusion that fluorination exerts a measurable electronic effect on the porphyrin core, despite the orthogonal orientation of the axial aryl groups with respect to the porphyrin plane.

To fully understand the reasons behind this and the electronic effects of adding fluorine atoms at the axial position, we conducted DFT studies, focusing on electron density behaviour through QTAIM analysis and polarization effects upon the successive addition.

### 2.3 Quantum Chemical Analysis

The Non‐Covalent Interaction (NCI) analysis,^[^ ^43^ ^]^ developed to identify and quantify weak non‐covalent interactions, is a valuable tool for understanding the reactivity and stability of interacting sites. NCI analysis employs the reduced density gradient (RDG) of the electron density to highlight regions of non‐covalent interactions through color‐coded 3D isosurfaces. These isosurfaces enable a qualitative evaluation of interaction types, typically with blue representing strong, attractive interactions (e.g., hydrogen bonding), green indicating weak van der Waals forces, and red signifying repulsive interactions (e.g., steric clashes). The method is further enhanced by incorporating the sign of the second eigenvalue (λ₂) of the Hessian matrix of the electron density, which helps differentiate between attractive and repulsive interactions. The method has been implemented recently in the NCI‐plot software version 4.2, which was utilized in this work.^[^ ^44^ ^]^

**Figure 3:**
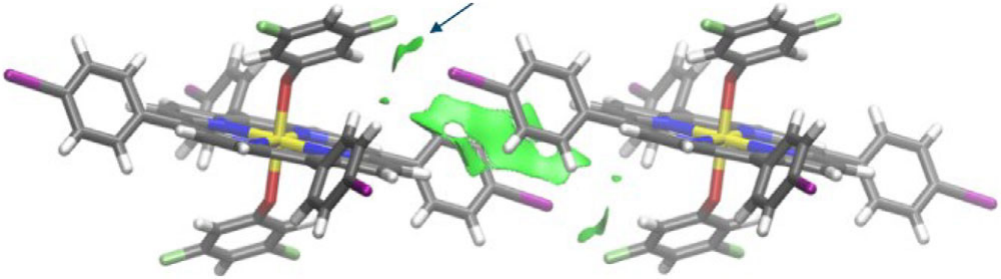
Reduced density gradient (RDG) isosurface (0.3 a.u.) representing the non‐covalent interactions between the I···F halogen bond‐ (indicated by dark blue arrow) and the π‐π interactions in the dimer containing two fluorine atoms in the axial position.

In this study, we focused solely on qualitative RDG analysis due to inconsistencies encountered during the calculations, as detailed in the Computational Methods Section in the Supporting Information. Specifically, a large external region appeared as weak interactions, masking the numerical results. Figure 3 highlights the key interactions in the dimer where two hydrogen atoms in each aromatic ring at axial positions were replaced by fluorine atoms, with the far region removed. Interestingly, the σ‐hole interaction (indicated by the dark blue arrow) was identified as weak rather than strongly attractive. Nonetheless, the small volume of the domain as well as its shape pin‐points towards directional character of the interaction. A key finding, apart from the previously discussed σ‐hole interactions, is the crucial role that *π*–*π* interactions between the aromatic rings play in stabilizing the dimer structure. This pattern is consistent across other dimers, as shown in the Supporting Information file (Figures S22 and S23), regardless of whether the RDG was computed using dimer wavefunctions or promolecular density. Given that numerical precision was not our primary focus due to the system's size, and promolecular density calculations are faster while still providing reliable qualitative insights as well as enabling us to remove intramolecular interactions, we opted for this method over using wavefunctions of the fragments.

**Scheme 2:**
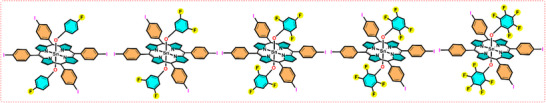
Constructed model structures of Sn(IV) porphyrin complexes used for computational evaluation of electronic effects, including dipole moments, atomic polarizabilities, and electrostatic potentials. Models were generated by modifying the geometry of complex **5** (fully fluorinated) and systematically removing fluorine atoms to create a full series of fluorination levels (from 1F to 5F).

To systematically investigate how axial fluorination influences electronic properties, particularly polarization and electrostatic potential, we aimed to analyze all five possible substitution levels (from mono‐ to penta‐fluorinated systems). While most variants were successfully obtained and structurally characterized, the crystal structure for the trisubstituted analogue could not be resolved experimentally. To address this limitation and ensure a consistent comparative framework, we constructed a complete computational model set using the following strategy: (i) the experimental geometry of complex **5**, featuring five fluorine atoms at the axial positions, was used as the structural template; (ii) fluorine atoms were sequentially replaced with hydrogen atoms to generate the 4‐F, 3‐F, 2‐F, and 1‐F substituted analogues, maintaining the core porphyrin geometry: (iii) each model (including 5‐F complex) was fully optimized at the DFT level, and the resulting structures served as the basis for calculating dipole moments, atomic polarizabilities, and electrostatic potential distributions. This modelling approach allows us to isolate the electronic effects of fluorination while preserving the structural framework of the porphyrin core. The resulting five‐model series is presented in Scheme 2. All computational details, including methods, basis sets, and convergence criteria, are provided in the ESI file.

The correlation between atomic dipole moments and the electrostatic potential (ESP) has been shown to be reliable for atoms that lack significant sigma‐hole character, such as fluorine atoms.^[^ ^45^, ^46^, ^47^, ^48^, ^49^ ^]^ In such cases, a higher dipole moment generally corresponds to an increased ESP. However, for atoms like iodine, which often feature pronounced sigma‐hole regions, a more accurate description of the ESP may require higher‐order multipolar expansions. To better capture the electrostatic properties in this study, we calculated the ESP for each monomeric structure using the same wavefunctions as those applied in the polarizability calculations. This consistent approach allowed for a more precise identification of ESP maxima and enabled a detailed correlation with dipole moments. Computational details are provided in the ESI.

**Figure 4:**
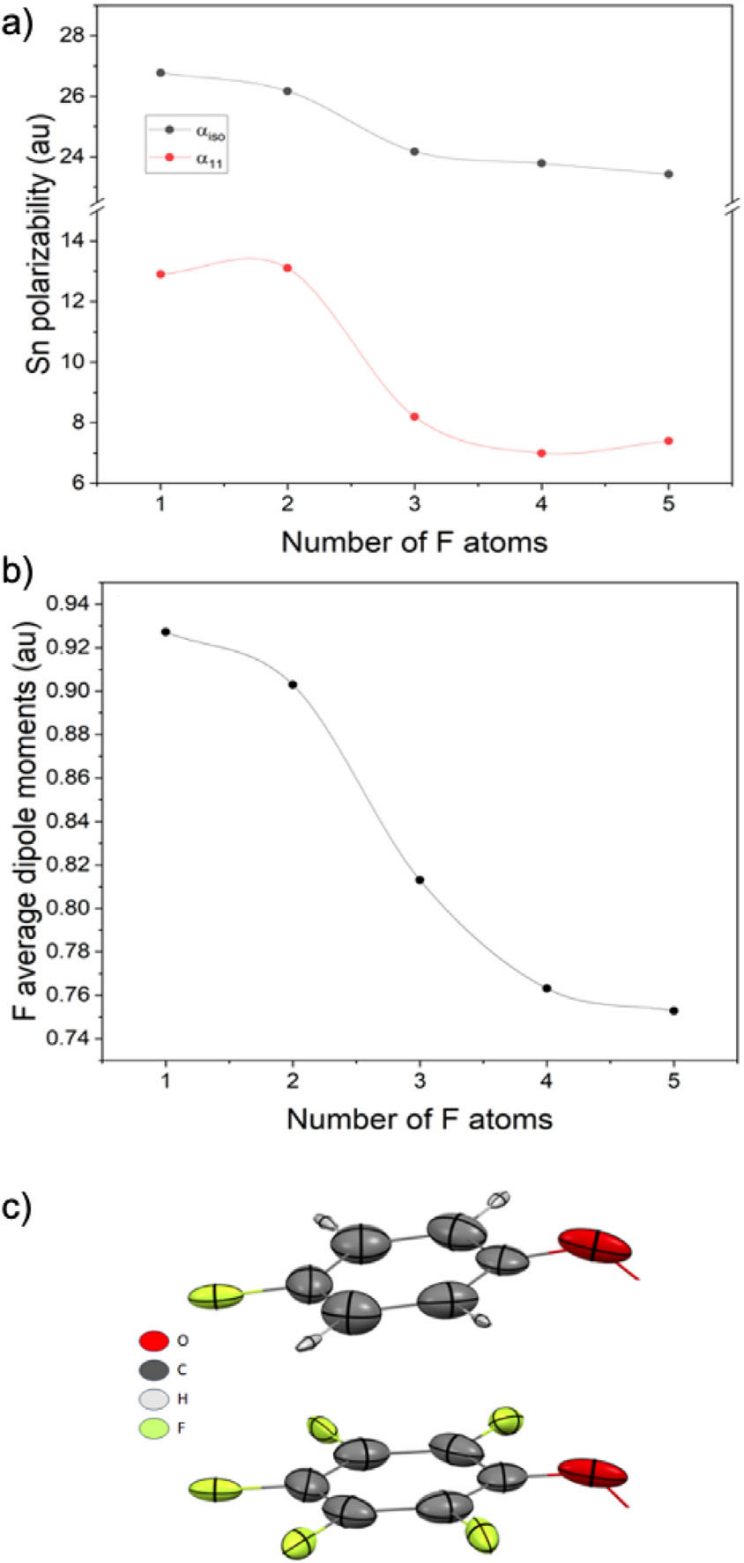
Dependence between the number of F‐atoms within the phenyl group and a) polarizability of Sn metal center and b) averaged dipole moments’ modules of fluorine atoms (right chart). *α_iso_* refers to isotropic polarizability, and *α*_11_ is the component of the polarizability tensor along the Sn─O bond. c) Representation of atomic polarizabilities in 1‐ (top) and 5‐fluorine substituted (bottom) phenyl ring. Ellipsoids are visualized with a scale factor of 0.02 Å^−2^.

Increased fluorination at the axial phenyl rings leads to greater local charge separation, particularly in C─F bonds, enhancing the dipole moment along those bonds. At the same time, this increased electrostatic character tends to localize electron density, thereby reducing polarizability, a well‐established trend in polar, less covalent bonds^[^ ^50^, ^51^ ^]^ The competition for electron density between fluorine substituents and the phenolate oxygen also alters the electronic environment of the Sn─O bond. As the number of fluorine atoms increases, we observe a consistent decrease in the polarizability of the tin center, especially along the Sn─O vector (Figure 4a), which we interpret as a consequence of weakened Sn─O bonding. This effect is further supported by the reorientation of polarizability tensors at the C─O bond. In less fluorinated systems, both carbon and oxygen ellipsoids are aligned toward the Sn center, indicative of stronger coordination. In contrast, in highly fluorinated systems, these tensors reorient toward one another, consistent with a redistribution of electronic density and a more localized, less polarizable bonding environment (Figure 4c).

Additionally, we observe that the dipole moments of fluorine atoms correlate with changes in the electrostatic potential across the molecule, particularly in the vicinity of iodine atoms where σ‐hole features emerge. These relationships, between dipole moments, polarizabilities, and ESP, highlight how electronic substitution at axial ligands modulates the electrostatic landscape of the porphyrin, even in the absence of direct conjugation between axial and equatorial positions. This interplay ultimately shapes the nature and directionality of sigma‐hole interactions observed in the solid state.

**Figure 5:**
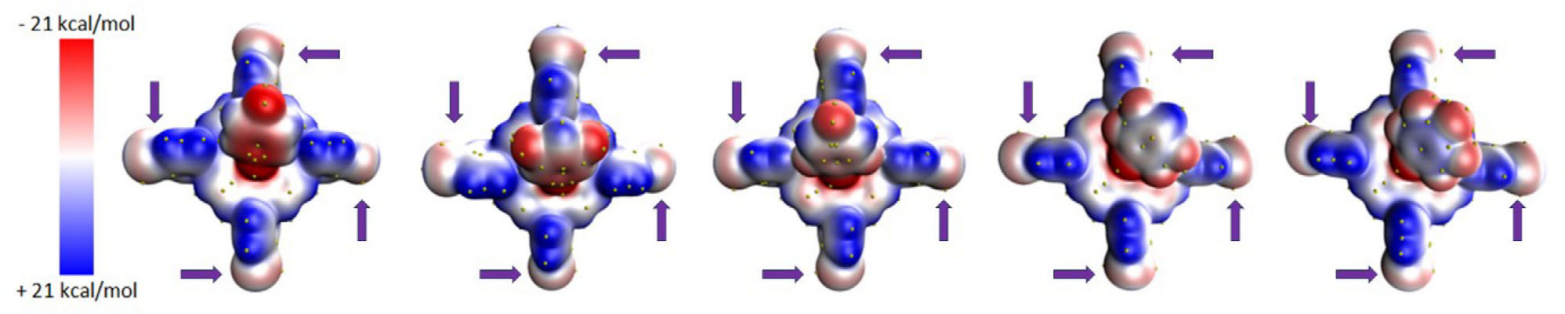
Electrostatic potential maps of porphyrin rings, plotted over an isoelectronic density surface at 0.001au, from one‐ (left) to five‐ (right) fluorine substituted aromatic ring. Yellow dots on isosurfaces represent the maxima of the electrostatic potential at the given isodensity value, and arrows indicate positions of iodine substituents.

**Figure 6:**
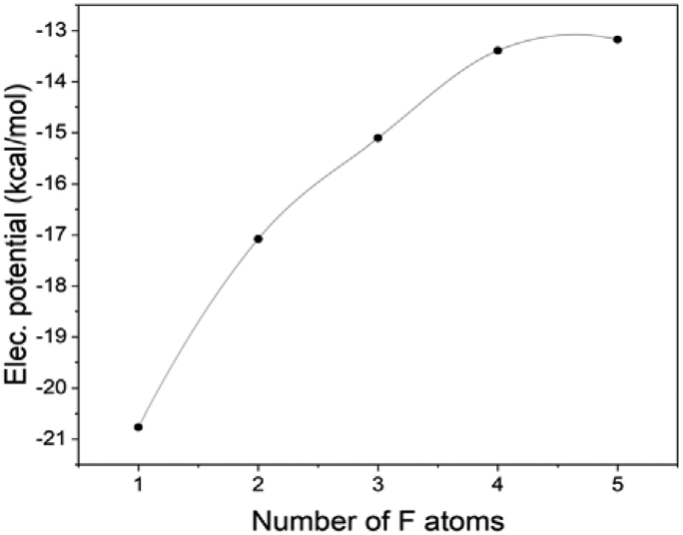
Correlation between the number of F‐atoms and the average electrostatic potential, calculated using its maxima values located over the 0.001 isodensity surface.

**Figure 7:**
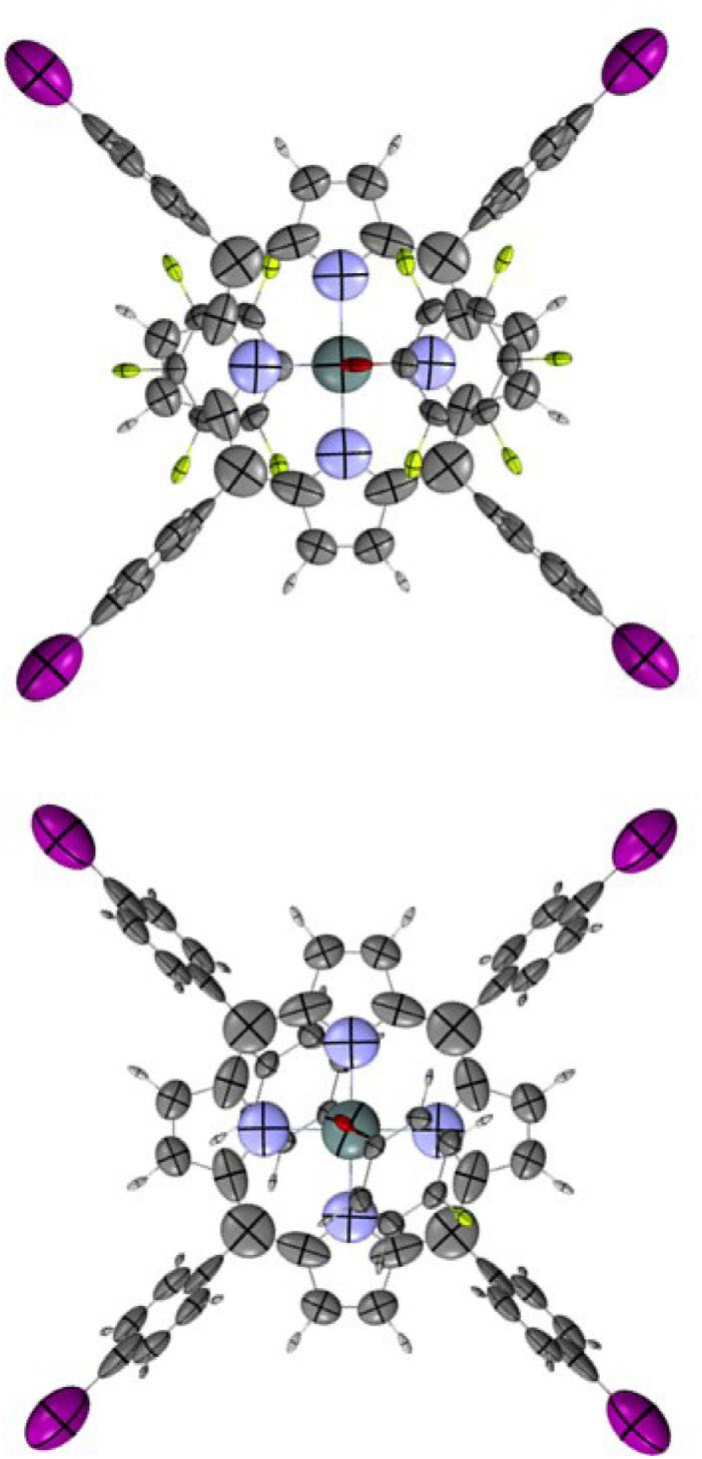
Distributed atomic polarizabilities of the mono‐substituted porphyrin ring (top) and its penta‐substituted offspring (bottom). The iodine‐substituted phenyl group is perpendicularly linked to the main ring, given the orthogonality of the atomic ellipsoids on the C─C bond connecting both fragments. The scaling factor for the polarizability ellipsoid is 0.2 Å^−2^.

As part of our analysis to establish a comprehensive link between the crystal structure and electro‐optical properties, we conducted a quantification of the electrostatic potential (ESP) at an isodensity surface (0.001au). Subsequently, we identified ESP critical points, revealing the presence of sigma‐hole potentials on the Iodine atoms, while no such potentials were observed over the fluorine atoms. These findings are visually depicted in Figure 5. With respect to fluorine atoms, the data presented in Figure 6 demonstrates a decrease in the absolute values of the electrostatic potential as the degree of substitution on the phenyl group increases. This observation is consistent with the correlation between dipole moments and ESP, which indicates that the absolute value of the potential tends to be higher when the dipole moment increases. Moreover, Figure 6 provides supporting evidence by illustrating a reduction in dipole moments of fluorine atoms with an increasing degree of substitution on the aromatic ring. The electrostatic potential for iodine atoms in the four extremities of the porphyrin plane, depicted in Figure 5, exhibits a division into a positive region on top of the atom and a negative halo around it, confirming sigma‐hole behaviour. One may notice that the increasing number of fluorine atoms does not significantly alter Iodine electrostatic potential. This observation can be attributed to the perpendicular orientation between the porphyrin ring and the external aromatic rings, which prevents resonance effects. It is consistent with the behaviour observed in the atomic polarizability tensors that also align perpendicularly to each other (see Figure 7). Consequently, the changes in polarizability of the tin metal centre have only a minor impact on the iodine atoms. Remarkably, all complexes exhibit the same behaviour regarding the orientation of the C‐C polarizabilities’ tensors, which is visualized in Figure 7.

The presence of a single fluorine atom attached to a phenyl ring leads to a proximity of positive potentials on iodine atoms (see Table 2) and negative potentials on fluorine atoms, promoting their interaction. However, it is important to note that the highest absolute potentials of F atoms are not directly located on top of the atom but exhibit a distinct halo‐like distribution over the fluorine. Consequently, the interactions between R‐F···I‐R are not linear but rather angular. The absolute potential of fluorine decreases to approximately 13 kcal/mol, reaching a level comparable to the absolute negative potential of iodine, around 8 kcal/mol. As a result, the electrostatic connection between I and F becomes less favourable as the number of F atoms increases. This observation explains why, despite the presence of multiple fluorine atoms in the 5‐substituted molecule, the I···F connections are not observed. Interestingly, although the F‐potential in the 4‐ and 5‐substituted molecules are relatively similar, for the former presence of a positively charged region arising from a hydrogen atom in the ring favours an arrangement where fluorine and hydrogen connect to (distinct) iodine atoms.

**Table 2:** Correlation between the degree of substitution of the aromatic rings and the sigma‐hole potential observed for the iodine atoms.

Number of F‐atoms	Average Positive Potential (kcal/mol)	Average Negative Potential (kcal/mol)
1	22.88	−7.2
2	23.13	−7.09
3	22.15	−7.89
4	21.92	−8.09
5	22.69	−7.41

## 3 Conclusion

In summary, we have isolated and characterized five [Sn(L)₂‐TIPP] porphyrin complexes featuring axially coordinated phenolate ligands with varying degrees of fluorination. Structural analysis reveals that changes in axial substitution significantly influence the supramolecular organization in the solid state, leading to diverse motifs such as ladder‐like arrangements and 2D cage‐like architectures. Electrochemical measurements demonstrate that increasing fluorine substitution induces consistent, though moderate, shifts in both oxidation and reduction potentials. These findings confirm that axial fluorination exerts a measurable electronic influence on the porphyrin core, despite the perpendicular orientation of the aryl ligands, which limits direct conjugation with the macrocycle. Computational studies further show that axial fluorination modulates local polarization effects, particularly at the phenolate oxygen and tin centers. Hirshfeld surface and NCI analyses indicate that halogen bonding motifs are geometrically preserved across the series, but theoretical analysis reveals subtle electronic changes that influence the electrostatic landscape. Most notably, axial fluorination alters dipole moments and polarizabilities in a predictable manner. Importantly, although the perpendicular orientation of the axial aryl ligands restricts resonance interaction with the porphyrin π‐system, fluorination induces inductive and field effects that propagate through the coordination framework. These effects influence the polarization of the Sn─O bond and modify local electrostatics, even if they do not significantly affect the sigma‐hole potential at the iodine atoms. The limited impact on sigma‐hole strength is likely a consequence of the spatial separation between iodine and the fluorinated groups.

Together, these experimental and theoretical results provide valuable insight into how localized electronic modifications at axial ligands influence both local bonding and non‐covalent interactions. This work highlights the need to distinguish between conjugative and field‐based effects in supramolecular systems and offers a framework for tuning self‐assembly behavior in halogen‐bonded metalloporphyrins.

## Supporting Information

The Supporting Information is available including synthetic details, experimental measurements and computational details for the studied complexes. The details of X‐ray structure refinements are given in ESI and the CCDC Deposition Numbers 2400902 (complex **1**); 2400905 (complex **2**); 2400904 (complex **3**); 2400903 (complex **4**); 2400901 (complex **5**) contain the supplementary crystallographic data for this paper. These data are provided free of charge by the joint Cambridge Crystallographic Data Centre and Fachinformationszentrum Karlsruhe Access Structures service. The authors have cited additional references within the Supporting Information.^[^ ^52^, ^53^, ^54^, ^55^, ^56^, ^57^, ^58^, ^59^, ^60^, ^61^, ^62^ ^]^

## Conflicts of Interest

The authors declare no conflicts of interest.

## Supporting information



Supporting Information

## Data Availability

The data that support the findings of this study are available in the supplementary material of this article.
