# Supplementary material for: Polarization and Dipole Moment Effects on Sigma‐Hole Potential in Tin(IV)‐Porphyrins
Source: Chemistry. 2025 Jul 22;31(45):e02099. doi: 10.1002/chem.202502099 (PMC12351427; doi:10.1002/chem.202502099)
Supplement: Supplementary file 1 — Supporting Information [file CHEM-31-e02099-s001.docx]

**Supporting Information**

**Polarization and Dipole Moment Effects on Sigma-hole Potential in tin(IV)-Porphyrins**

Rafia Siddiqui,^[a]^ Raphael F. Ligorio,^[b]^ Hatem, M. Titi,^[c]^ Sushil Kumar Pandey,^[d]^ Anna Krawczuk^[b]^* and Ranjan Patra^[a]^*

[a] Ms. R.Siddiqui and Dr. R. Patra.,
Amity Institute of Click Chemistry Research and Studies,

Amity University, Noida, Uttar Pradesh, India.
E-mail: [rpatra@amity.edu](mailto:rpatra@amity.edu)

[b] Mr. R. F. Ligorio and Prof. A. Krawczuk
Institute of Inorganic Chemistry
University of Göttingen
Tammannstrasse 4, D-37077 Göttingen, Germany

E-mail: [anna.krawczuk@uni-goettingen.de](mailto:anna.krawczuk@uni-goettingen.de)

[c] Dr. H. M. Titi
Department of Chemistry
McGill University
801 Sherbrooke St. West, Montreal, QCH3A0B8, Canada.

[d] Dr. S. K. Pandey
Aragen Life Sciences Ltd.
Mallapur, Secunderabad, Telangana-500076, India

**EXPERIMENTAL SECTION**

**Materials:** Pyrrole, 4-iodobenzaldehyde, 2,3-diflouorophenol, 2,4-diflouorophenol, 3,5- diflouorophenol, 2,3,4,5-tetrafluorophenol, 2,3,4,5,6-pentafluorophenol were obtained from TCI chemicals. Solvents like propionic acid, chloroform, dichloromethane (DCM), methanol, acetone, dimethylformamide (DMF), and hexane were obtained from commercial sources and purified by standard procedures before use. Free-base porphyrins were prepared following the Adler method.^[52]^ Sn(TIPP)(OH)_2_ were prepared by literature methods.^[53]^

**Experimental Measurements:**

UV-vis spectra were recorded on a Perkin Elmer UV-Vis spectrometer. IR spectra were recorded on a Bruker Tensor 27 system spectrophotometer in ATR mode. Time-resolved fluorescence decays were collected with a commercial time-correlated single-photon counting (TCSPC) setup (Life Spec II, Edinburgh Instruments). The steady-state fluorescence spectra of all the samples were recorded using Perkin Elmer LS 55 fluorescence spectrometer. The samples were prepared in dry dichloromethane and the concentration was adjusted so that the absorbance was less than 0.1. Absorption correction was done to normalize the data. Elemental (C, H, and N) analyses were performed on a Perkin-Elmer 2400II elemental analyzer.

Powder X-ray Diffraction (PXRD) was measured by a XEUSS SAXS/WAXS system by Xenocs, operated at 50 kV and 0.60 mA. The X-ray radiation was collimated with FOX2D mirror and two pairs of scatter less slits from Xenocs. The data were collected in the transmission mode geometry using Cu Kα radiation (wavelength λ = 1.54 Å). The fibre diagrams were recorded using an image plate system (Mar 345 detector) and processed using Fit2D software.

**Scheme S1**: Schematic illustration of the Sn(IV)-5,10,15,20-*meso*-tetrakis(4-iodophenyl)porphyrin scaffolds used in this study. Axial ligands (L) 2,3-Difluoro Phenol (**1**), 2,4-Difluoro Phenol (**2),** 3,5-Difluoro Phenol (**3**), 2,3,5,6-Tetrafluoro Phenol (**4**), 2,3,4,5,6 -Pentafluoro Phenol (**5**) which was structurally characterized through single crystal X-ray diffraction.

**General procedure of synthesis of the complexes:**

A mixture of trans-dihydroxo[5,10,15,20-tetrakis(4-iodophenyl)porphyrinato]tin(IV) (0.01 mmol) was dissolve in 5 ml of CHCl_3_ and corresponding halogenated phenol (0.025 mmol) was dissolve in 0.5 mL of DMF. The resulting solution was heated for one hour at 70 °C in a bath reactor. After cooling, the mixture was left for slow evaporation at ambient conditions dark red solid was precipitated out. Filter the solid and wash it with hexane. All the complexes were isolated around 60-70% yield.

**Complex 1.** X-ray quality crystals were obtained by slow evaporation of DMF-CHCl_3_ (1:10) solution of **1** into the hexane. After 7 days fine pink crystal of complex was obtained. Yield (7.9 mg, 78%). FT-IR (KBr, cm^-1^) 1656, 1432, 1225, 1042, 782, 754, 645, 543. UV-Vis in DCM: λmax/nm (log e) 422(5.46), 552(2.64), 585(2.12).

**Complex 2.** X-ray quality crystals were obtained through slow evaporation of the DMF-CHCl_3_ (1:10) solution of **complex 2**. After 7 days fine pink crystal of complex was obtained. Yield (7.9 mg, 76%). FT-IR (KBr, cm^-1^) 1646,, 1443, 1234, 1061, 747, 732, 648, 545. UV-Vis in DCM: λmax/nm (log e) 423(5.42), 554(2.88), 586 (2.13).

**Complex 3.** X-ray quality crystals were obtained through slow diffusion of the CHCl_3_ solution of complex **3** into the diethyl ether. After 10 days fine pink crystal complex was obtained. Yield (7.9 mg, 75%). FT-IR (KBr, cm^-1^) 1645, 1429, 1212, 1046, 776, 732, 654, 546. UV-Vis in DCM: λmax/nm (log e) 424(5.38), 556(2.78), 581(2.11).

**Complex 4.** X-ray quality crystals were obtained after a slow evaporation of the CHCl_3_ solution of complex **4**. After 8-9 days fine pink crystal of the complex was obtained. Yield (7.9 mg, 78%). FT-IR (KBr, cm^-1^) 1672, 1447, 1221, 1065, 787, 756, 642, 565, UV-Vis in DCM: λmax/nm (log e) 423(5.52), 550(2.66, 587(2.11).

**Complex 5.** X-ray quality crystals were obtained after slow evaporation of CHCl_3_ solution of complex **5**. After 7-8 days fine pink crystal of complex was obtained. Yield (7.9 mg, 78%). FT-IR (KBr, cm^-1^) 1702, 1443, 1212, 1056, 776, 762, 648, 565. UV-Vis in DCM: λmax/nm (log e) 424(5.68), 554(2.98), 586(2.12).

**Single Crystal X-ray Structure Determination.**

X-ray Structure Determination. Single-crystal X-ray diffraction data for complexes **1**−**5** were collected with a “SuperNova diffractometer” equipped with a HyPix3000 detector from Rigaku Oxford Diffraction equipped with Mo*Kα* (0.71073 Å) at 293(2) K. Data collection and reduction were performed with an inbuilt program suite (CrysAlisPro 1.171.39.33c (Rigaku OD, 2017)), and an absorption correction (multiscan method) was also done. Structures were solved by the direct method using SHELXS-97^[54]^ and were refined on F^2^ using the full-matrix least-squares technique using the SHELXL-2018/3^[55]^ program package on the WINGX^[56]^ platform. All non-hydrogen atoms were refined anisotropically. Hydrogen atoms were fixed at their stereochemical positions and were refined using a riding model with the isotropic displacement parameters U_iso_ constrained to 1.2 U_eq_ of their parent carbon atoms. Most of the complex solvents are present in the unit cell except complex **5**.

In complex **1**, a disordered CHCl_3_ solvent molecule was identified and modelled using the **PART** instruction. In complex **2**, the fluorine atoms in the ortho positions exhibit positional disorder, refined with 50% occupancy, and the structure also includes disordered DMF solvent molecules.
Complex **3** contains a disordered CHCl₃ solvent molecule within the unit cell.
In complex **4**, a disordered cyclohexane molecule is located within the porphyrin cavity. This disorder was modelled using the **PART** and **DFIX** commands.

##

## Table S1. Crystal Data and Data Collection Parameters

| CCDC No | **2400902** | **2400905** | **2400904** | **2400903** | **2400901** |
| --- | --- | --- | --- | --- | --- |
| Formula | C_57_H_31_C_l3_F_4_I_4_N_4_O_2_Sn | C_61_H_36_C_l6_F_4_I_4_N_5_O_3_Sn | C_57_H_31_C_l3_F_4_I_4_N_4_O_2_Sn | C_68_H_50_F_8_I_4_N_4_O_2_Sn | C_56_H_24_F_10_I_4_N_4_O_2_Sn |
| Formula weight | 1612.52 | 1801.96 | 1612.52 | 1733.43 | 1601.10 |
| Colour | Purple | Purple | Purple | Purple | Purple |
| Crystal system | Monoclinic | Monoclinic | Triclinic | Triclinic | Monoclinic |
| Space group | *C*2/c | *C*2/c | *P*-1 | *P*-1 | *C*2/c |
| *a*, Å | 27.1423(7) | 25.5293(5) | 8.9763(5) | 8.1313(1) | 24.1935(6) |
| *b*, Å | 9.4408(3) | 9.8953(2) | 10.8693(3) | 12.8318(2) | 8.1958(2) |
| *c*, Å | 25.3912(5) | 25.1632(4) | 15.0986(4) | 15.2765(3) | 29.1784(7) |
| *α* , deg | 90 | 90 | 99.954(2) | 101.340(2) | 90 |
| *β*, deg | 106.990(2) | 102.021(2) | 96.827(3) | 95.289(1) | 111.666(3) |
| *γ*, deg | 90 | 90 | 97.759(3) | 99.230(1) | 90 |
| V, Å3 | 6222.4(3) | 6217.3(2) | 1422.31(10) | 1529.93(4) | 5376.9(3) |
| Z | 4 | 4 | 1 | 1 | 4 |
| dcalcd, g•cm-3 | 1.721 | 1.925 | 1.883 | 1.881 | 1.978 |
| μ, mm-1 | 2.577 | 2.717 | 2.819 | 2.510 | 2.853 |
| *F*(000) | 3064 | 3444 | 766 | 836 | 3024 |
| No. of unique data | 5494 | 5482 | 5006 | 5407 | 4741 |
| No. of params. refined | 354 | 387 | 354 | 389 | 349 |
| GOF on F^2^ | 1.055 | 1.056 | 1.064 | 1.077 | 1.057 |
| R1a [*I*> 2σ(*I*)] | 0.0491 | 0.0388 | 0.0458 | 0.0521 | 0.0326 |
| R1^a^ (all data) | 0.0588 | 0.0472 | 0.0606 | 0.0604 | 0.0366 |
| wR2b (all data) | 0.1613 | 0.1234 | 0.1390 | 0.1535 | 0.1060 |

## Table S2. Selected Bond Distance and Angles taken from single crystal X-ray diffraction experiments (# represents equivalent position).

| **Bond Distances (Å)** | **Complex 1** | **Complex 2** | **Complex 3** | **Complex 4** | **Complex 5** |
| --- | --- | --- | --- | --- | --- |
| Sn1-N1 | 2.079(6) | 2.083(3) | 2.088(4) | 2.094(4) | 2.086(2) |
| Sn1-N2 | 2.084(6) | 2.102(3) | 2.087(4) | 2.078(4) | 2.095(3) |
| Sn1-N1# | 2.079(6) | 2.083(3) | 2.088(4) | 2.094(4) | 2.086(2) |
| Sn1-N2# | 2.084(6) | 2.102(3) | 2.087(4) | 2.078(4) | 2.095(3) |
| Sn1-O1 | 2.068(6) | 2.071(3) | 2.036(3) | 2.068(4) | 2.076(2) |
| Sn1-O1# | 2.068(6) | 2.071(3) | 2.036(3) | 2.068(4) | 2.076(2) |
| **Bond Angles (°)** | | | | | |
| N1-Sn1-N1# | 180.0 | 180.0 | 180.0 | 180.0 | 180.0 |
| N1-Sn1-N2 | 90.4(2) | 90.27(14) | 89.86(15) | 90.09(16) | 89.06(10) |
| N1#-Sn1-N2 | 89.6(2) | 89.73(2) | 89.87(15) | 89.91(16) | 90.94(10) |
| N1#-Sn1-N2# | 90.4(2) | 90.27(14) | 90.13(15) | 90.09(16) | 89.06(10) |
| N1-Sn1-N2# | 89.6(2) | 89.73(14) | 89.86(15) | 89.91(16) | 90.94(10) |
| N2#-Sn-N2 | 180.00(2) | 180.00(14) | 180.00(15) | 180.00(16) | 180.00(15) |
| O1-Sn1-N1 | 90.4(2) | 89.64(14) | 92.22(15) | 89.41(16) | 88.71(10) |
| O1-Sn1-N1# | 89.6(2) | 90.36(14) | 87.78(15) | 90.59(16) | 91.29(10) |
| O1#-Sn1-N1 | 89.6(2) | 89.64(14) | 87.78(15) | 90.59(16) | 91.29(10) |
| O1#-Sn1-N1# | 90.4(2) | 89.64(14) | 92.22(15) | 89.41(16) | 88.71(10) |
| O1#-Sn1-N2 | 89.9 (3) | 88.69(14) | 90.09(15) | 91.67(15) | 90.57(11) |
| O1#-Sn1-N2# | 89.9 (3) | 88.69(14) | 90.09(15) | 88.33(15) | 89.43(11) |
| O1-Sn1-N2 | 91.0 (3) | 91.31(14) | 89.91(15) | 88.33(15) | 89.43(11) |
| O1-Sn1-N2# | 91.0 (3) | 91.31(14) | 89.91(15) | 91.67(15) | 90.57(11) |
| O1-Sn1-O1# | 180.0 | 180.0 | 180.0 | 180.0 | 180.0 |


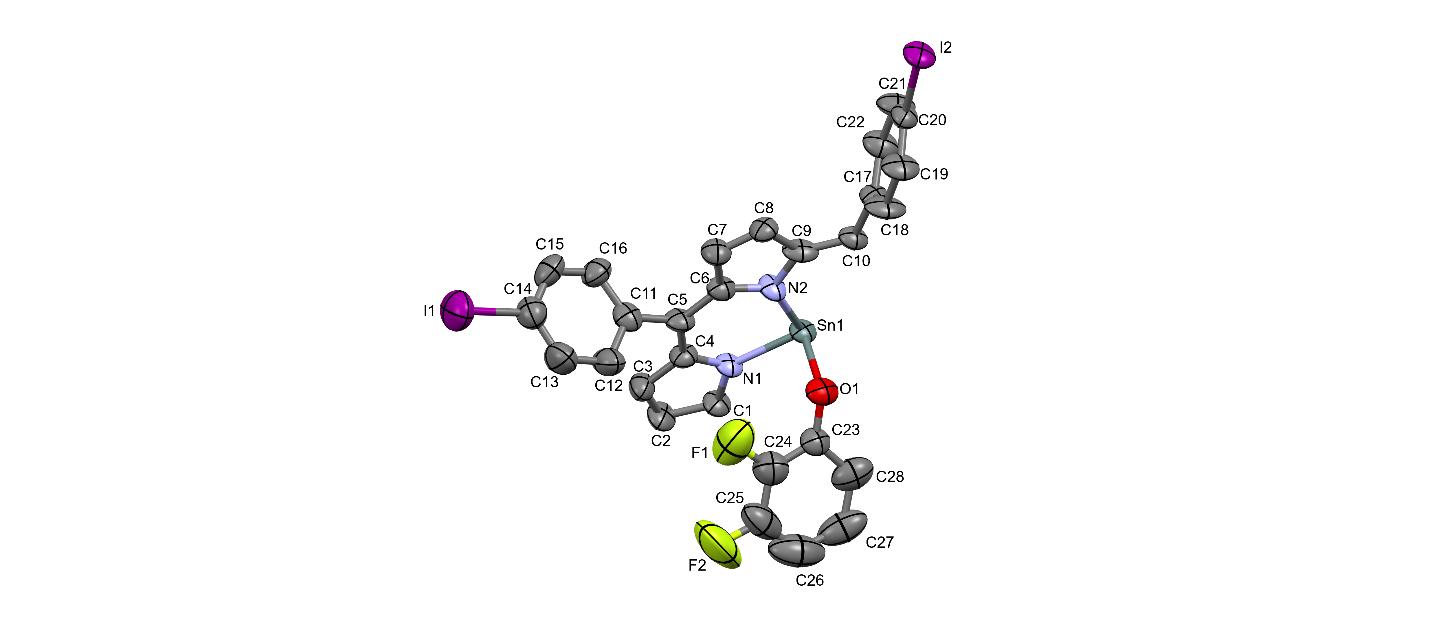

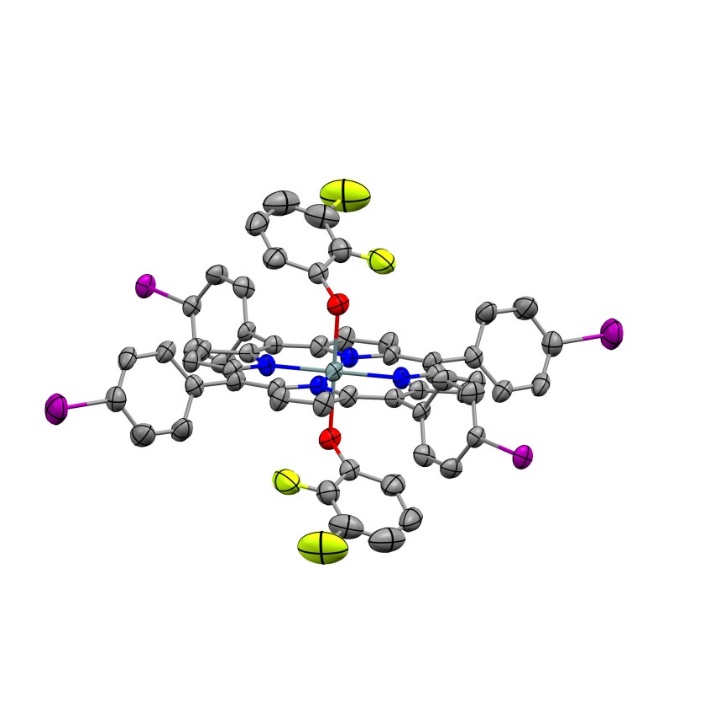


**Figure S1.** Asymmetric unit of complex **4** with labelling scheme (left) and perspective view (right) showing 40% thermal ellipsoids for all non-hydrogen atoms at 293 K (solvent molecules and H-atoms have been omitted for clarity).


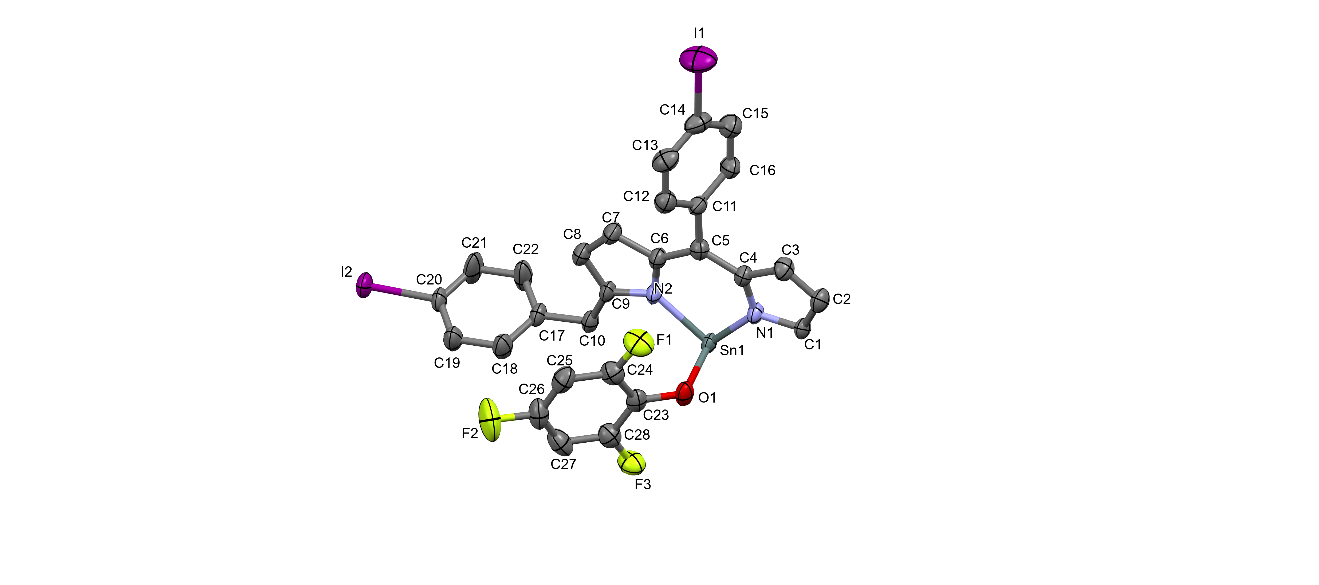

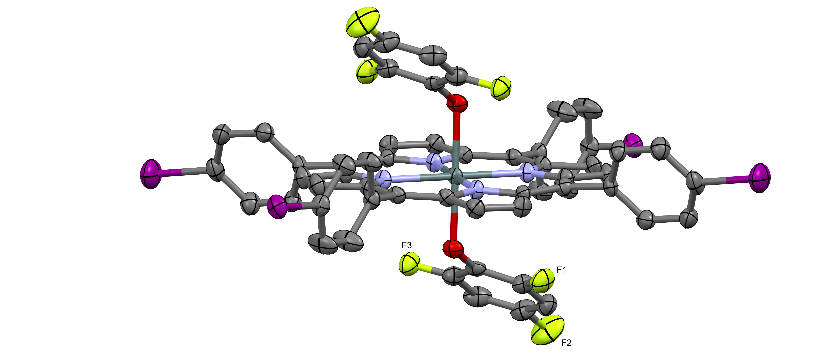


**Figure S2.** Asymmetric unit of complex **4** with labelling scheme (left) and perspective view (right) showing 40% thermal ellipsoids for all non-hydrogen atoms at 293 K (solvent molecules and H-atoms have been omitted for clarity). Fluorine atoms F1 and F3 are included in the asymmetric unit, however they exhibit occupational disorder with occupancy 0.5 on each site.


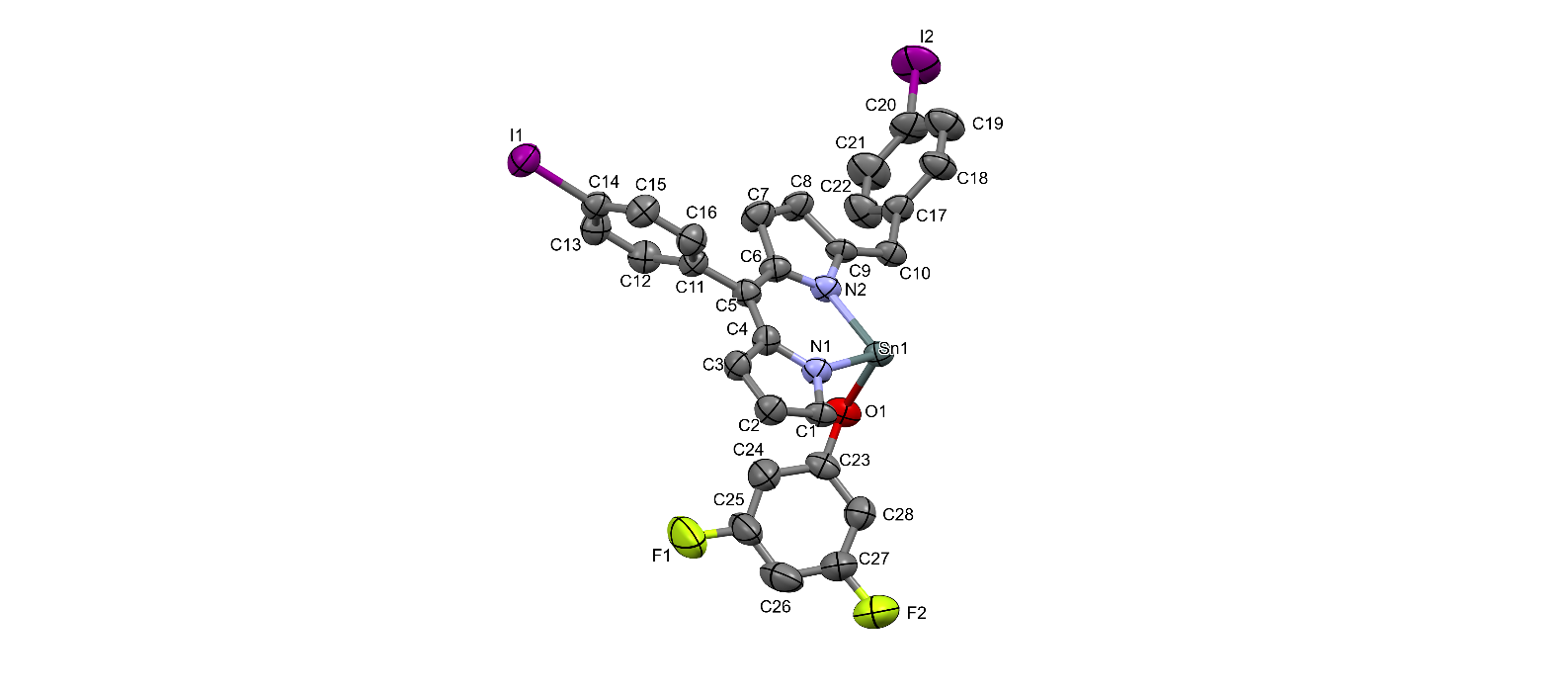

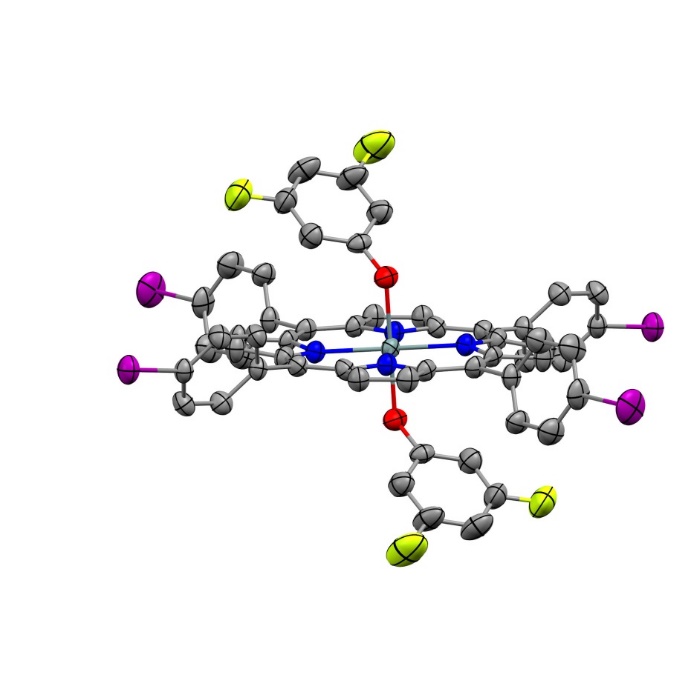


**Figure S3.** Asymmetric unit of complex **3** with labelling scheme (left) and perspective view (right) showing 40% thermal ellipsoids for all non-hydrogen atoms at 293 K (solvent molecules and H-atoms have been omitted for clarity).


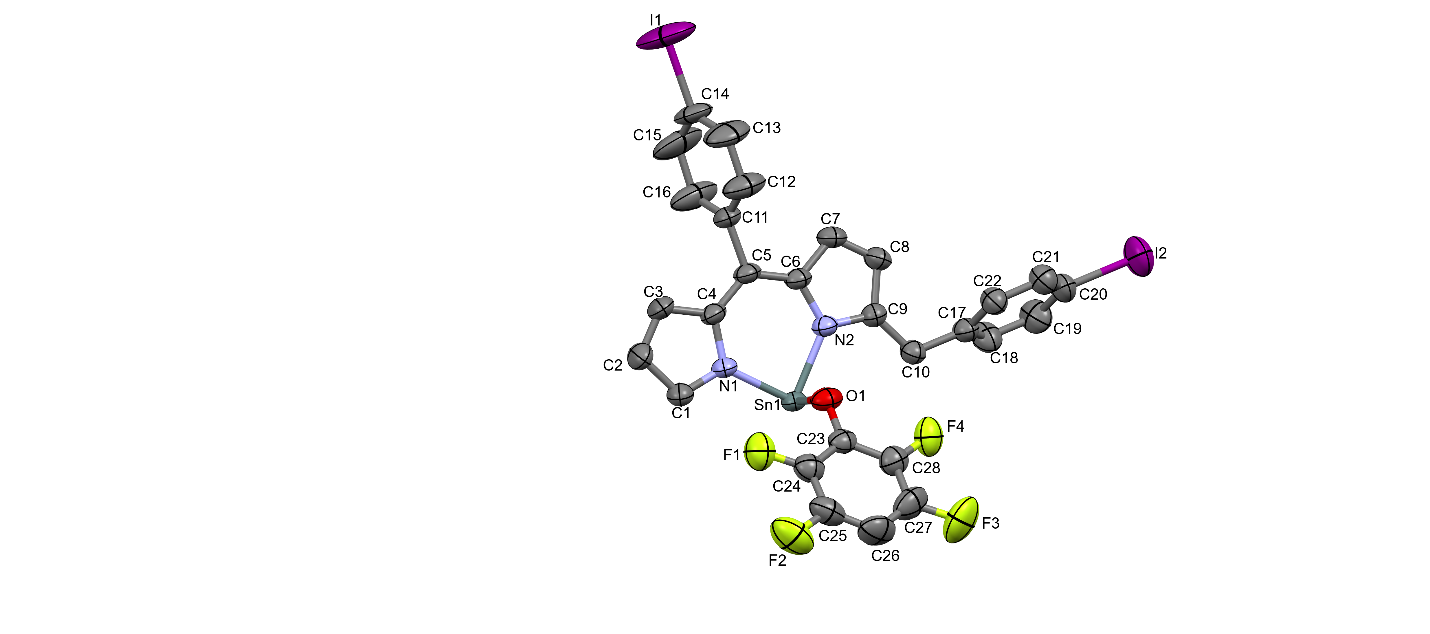

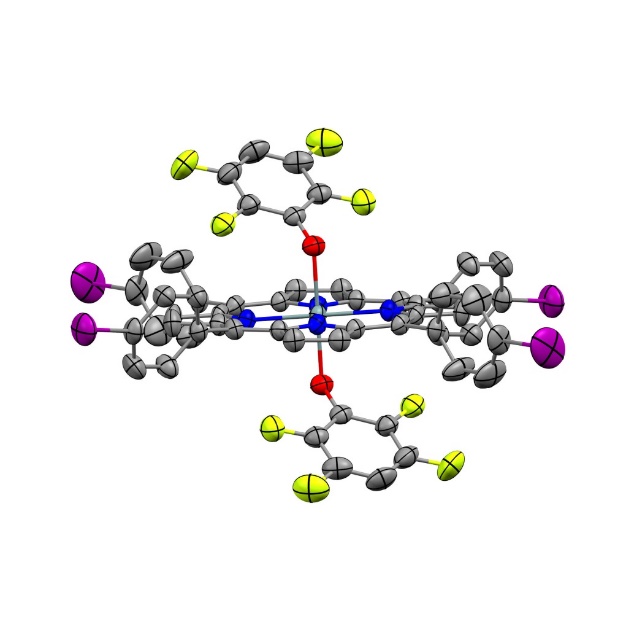


**Figure S4.** Asymmetric unit of complex **4** with labelling scheme (left) and perspective view (right) showing 40% thermal ellipsoids for all non-hydrogen atoms at 293 K (solvent molecules and H-atoms have been omitted for clarity).


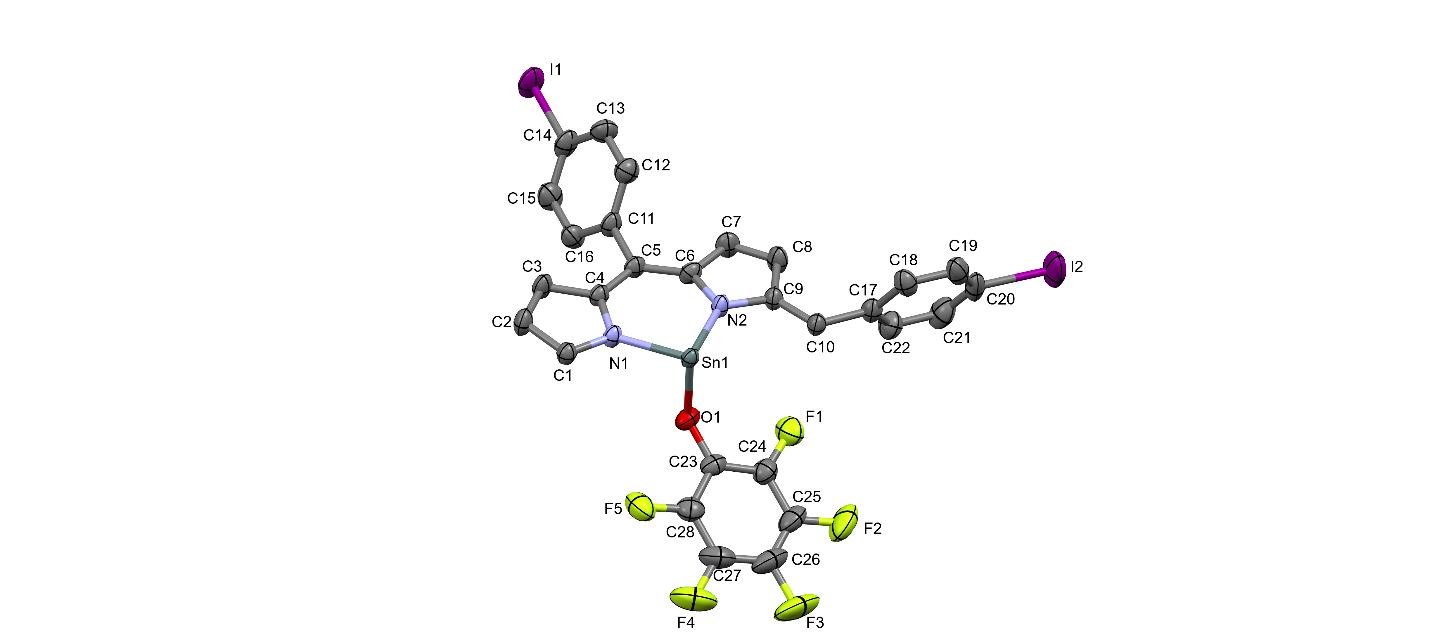

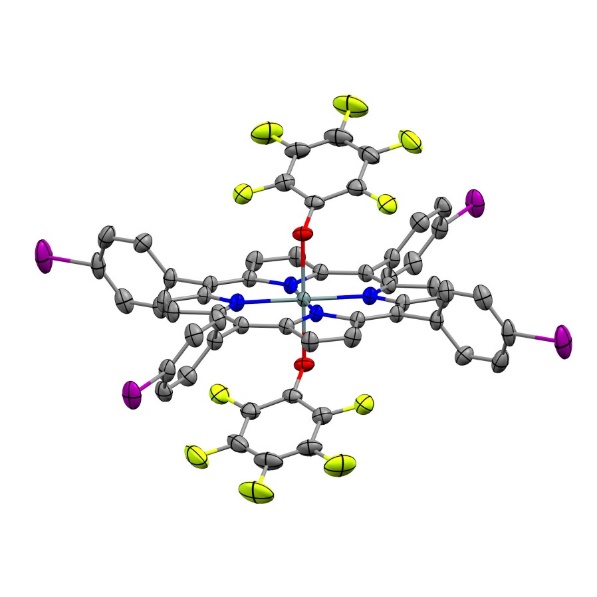


**Figure S5.** Asymmetric unit of complex **5** with labelling scheme (left) and perspective view (right) showing 40% thermal ellipsoids for all non-hydrogen atoms at 293 K (H-atoms have been omitted for clarity).


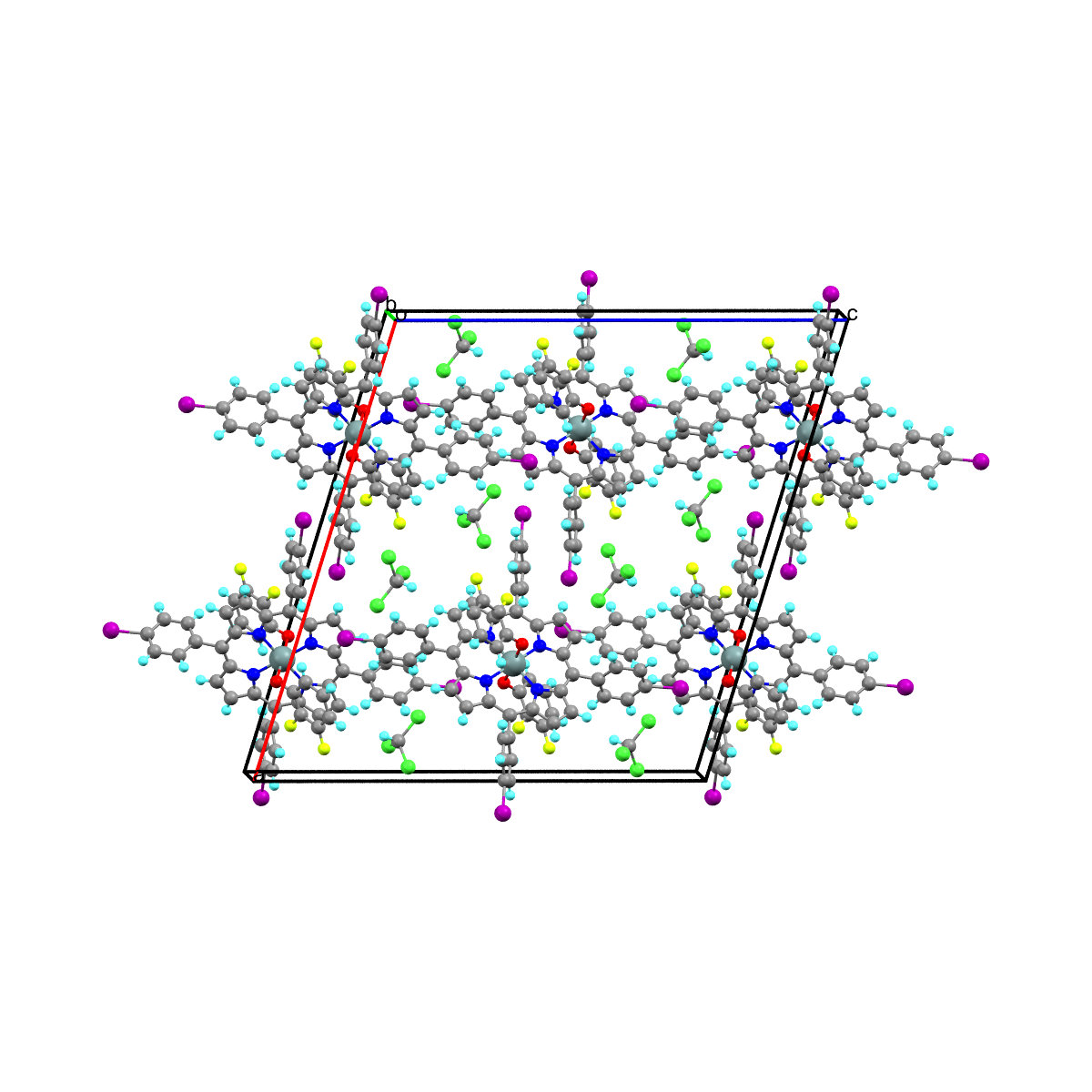


**Figure S6.** Perspective view of complex **1** (H-atoms have been omitted for clarity).


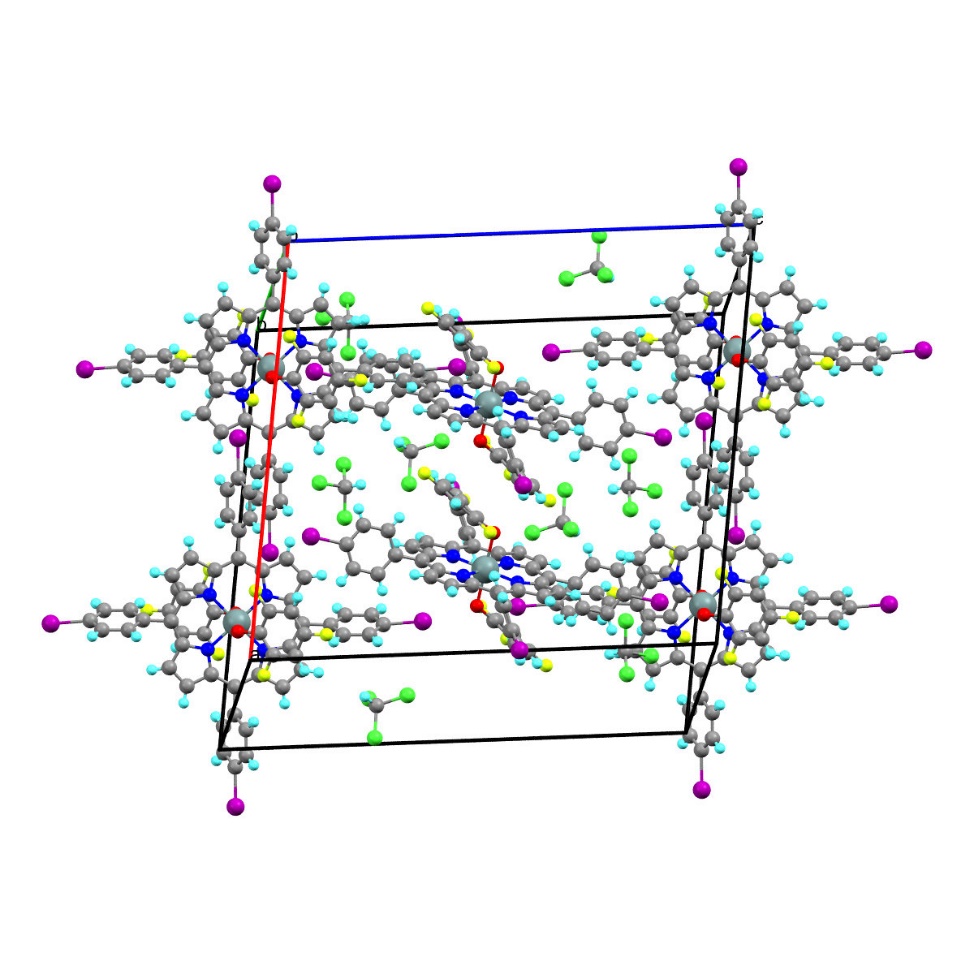


**Figure S7.** Perspective view of complex **2** (H-atoms have been omitted for clarity).


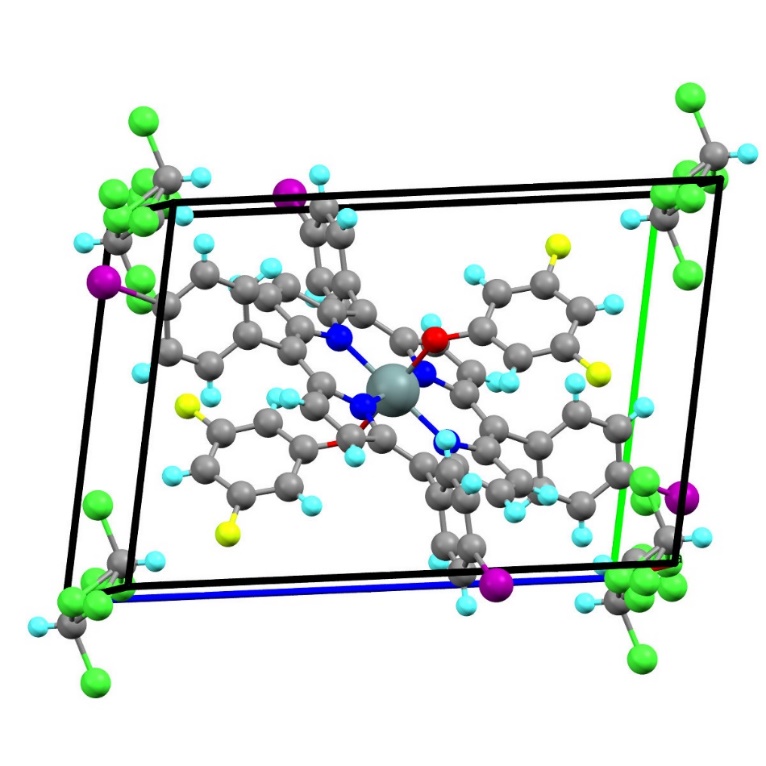


**Figure S8.** Perspective view of complex **3** (H-atoms have been omitted for clarity).


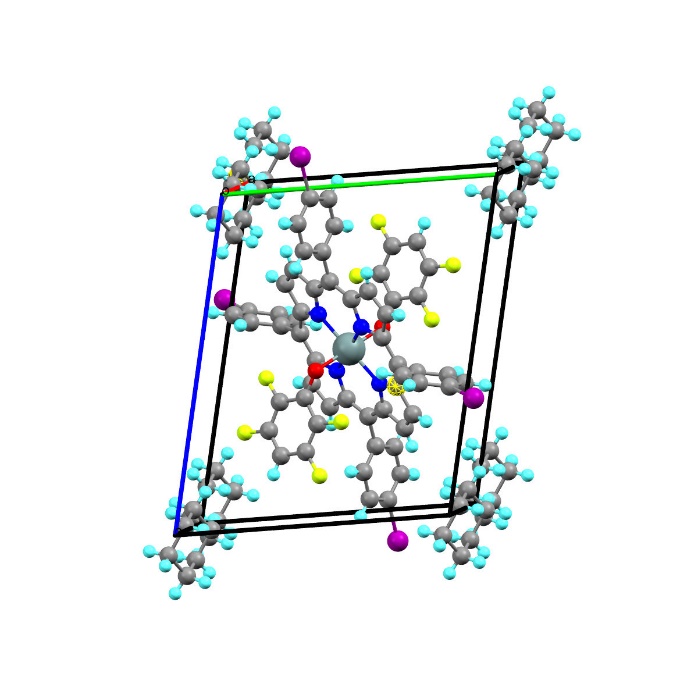


**Figure S9.** Perspective view of complex **4** (H-atoms have been omitted for clarity).


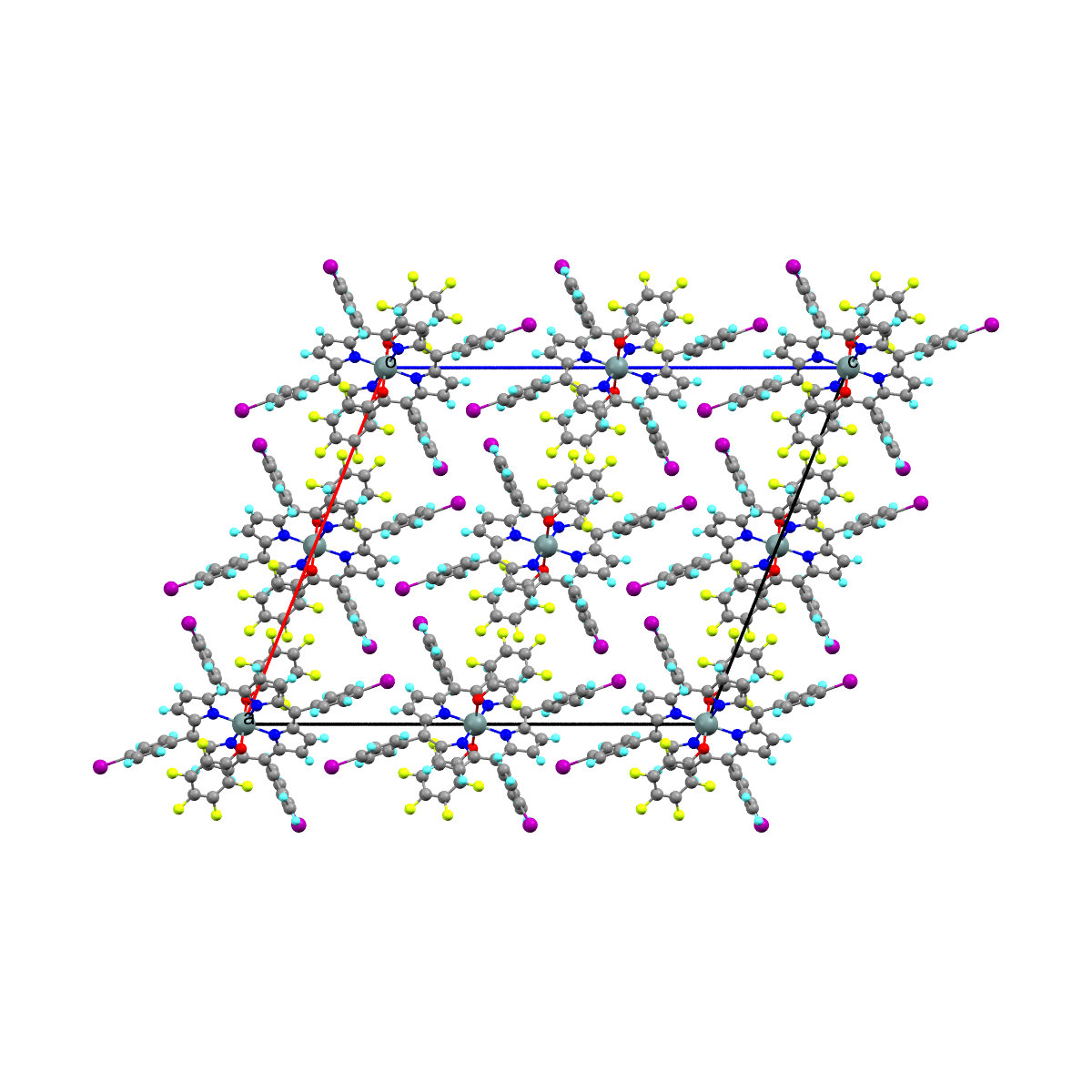


**Figure S10.** Perspective view of complex **5** (H-atoms have been omitted for clarity).


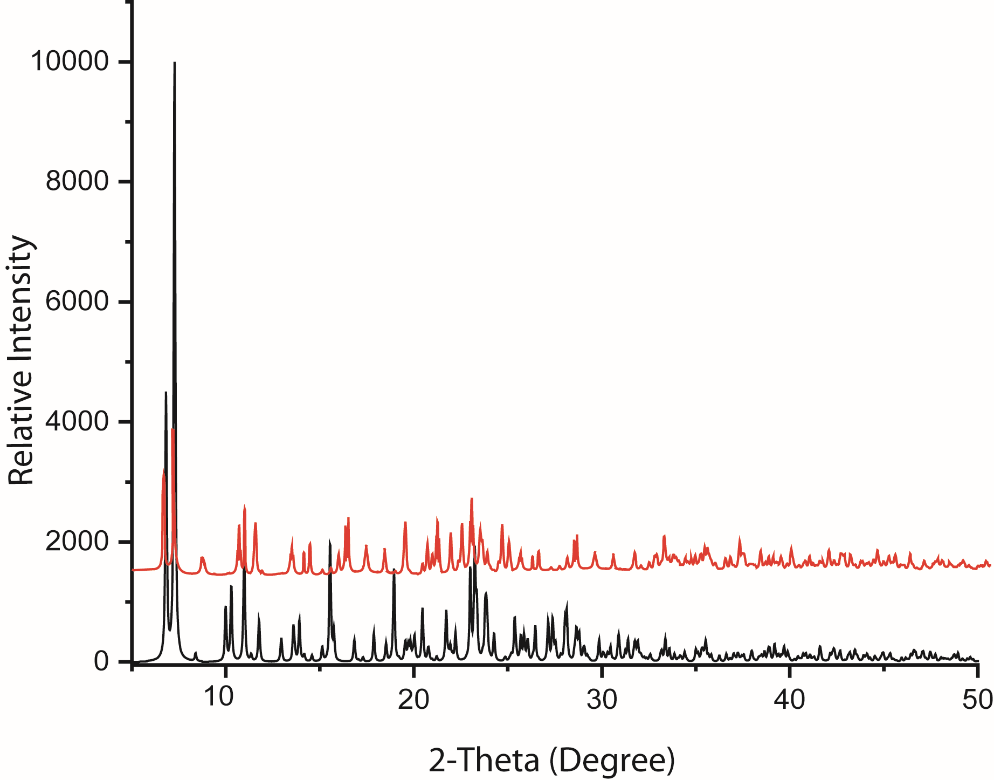


**Figure S11**. Overlapped experimental (red) and simulated (black) PXRD pattern of complex **1** at 298K**.** Simulated spectra (black) obtained from single crystal structure (Mercury software).


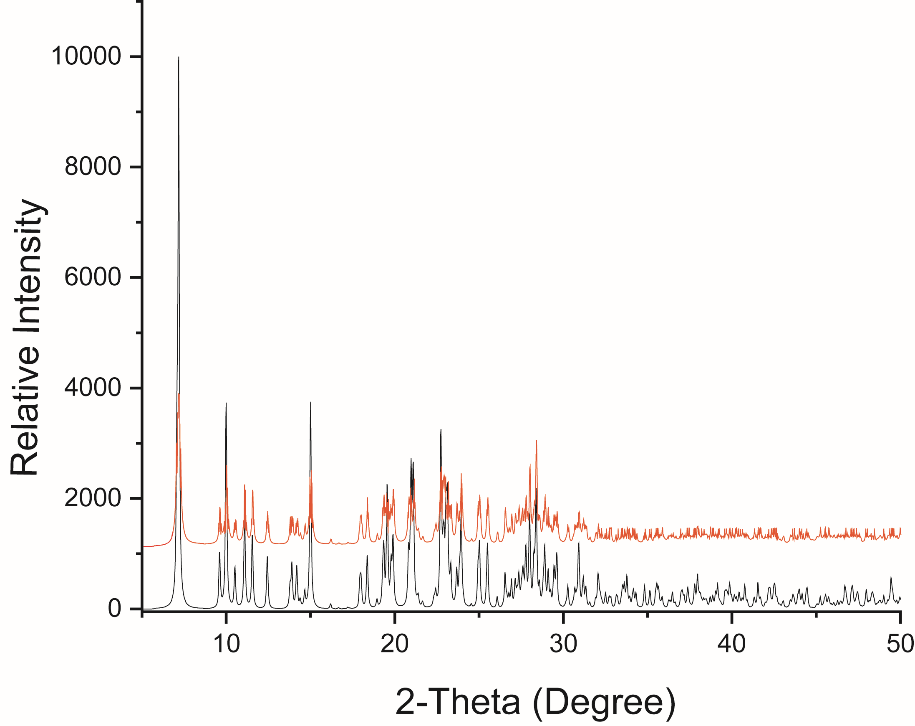


**Figure S12**. Overlapped experimental (red) and simulated (black) PXRD pattern of complex **2** at 298K**.** Simulated spectra (black) obtained from single crystal structure (Mercury software).


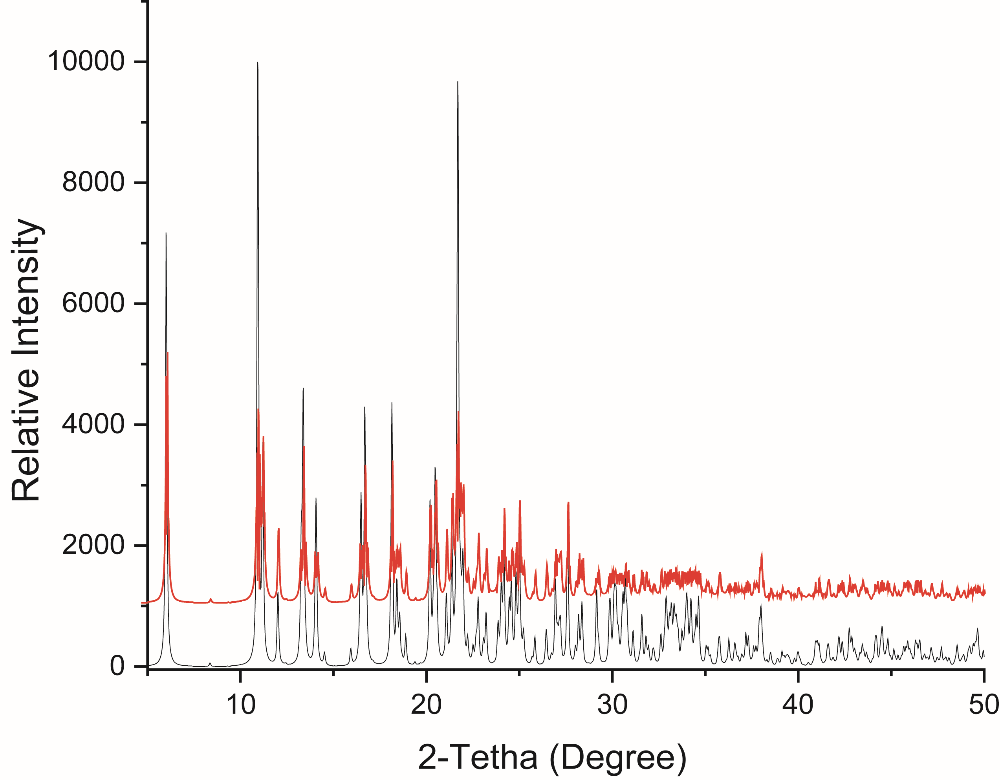


**Figure S13**. Overlapped experimental (red) and simulated (black) PXRD pattern of complex **3** at 298K**.** Simulated spectra (black) obtained from single crystal structure (Mercury software).


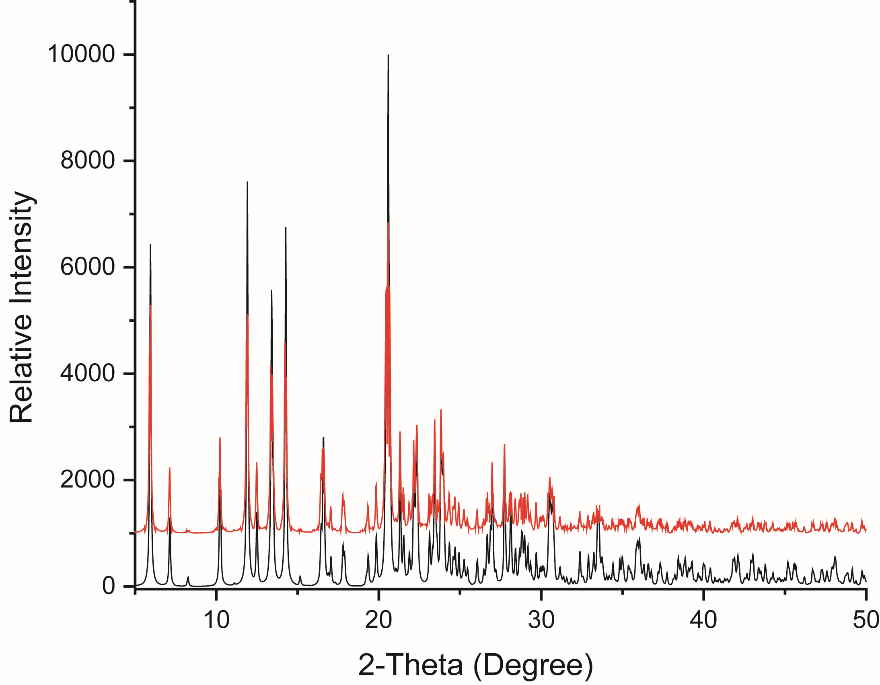


**Figure S14**. Overlapped experimental (red) and simulated (black) PXRD pattern of complex **4** at 298K**.** Simulated spectra (black) obtained from single crystal structure (Mercury software).


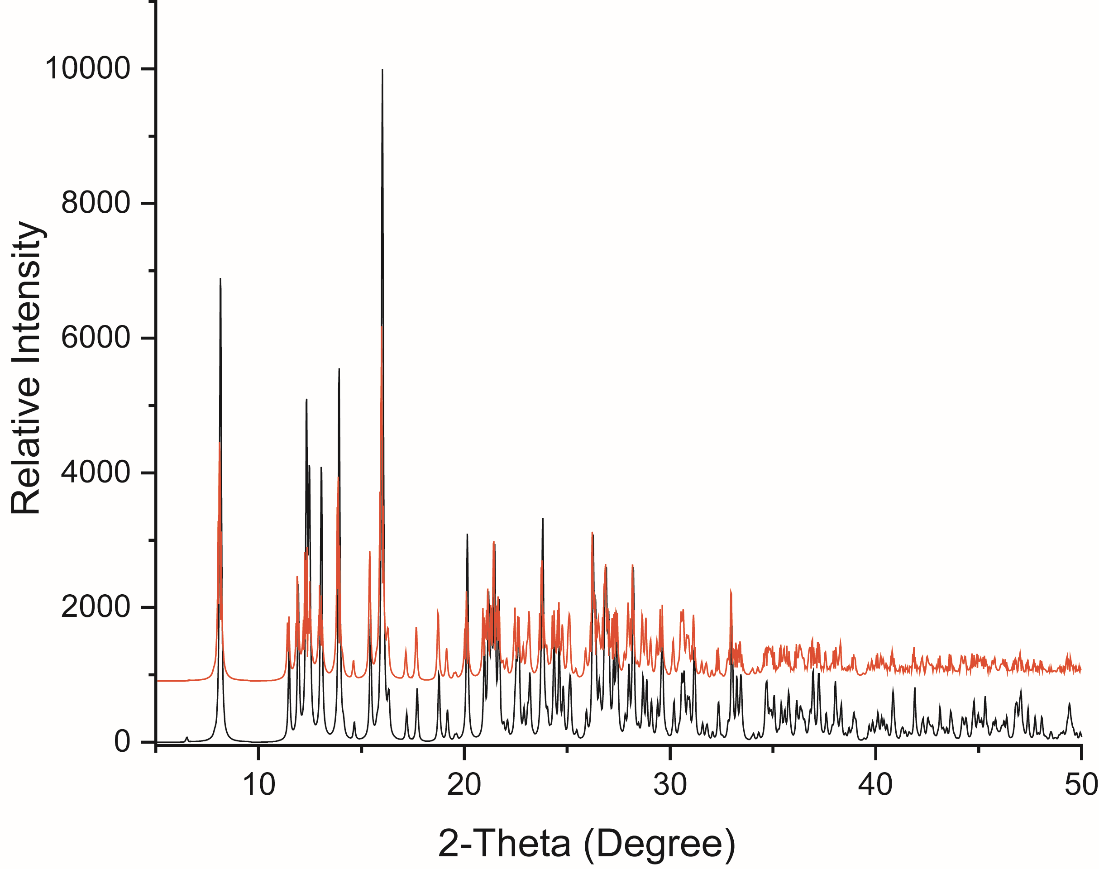


**Figure S15**. Overlapped experimental (red) and simulated (black) PXRD pattern of complex **5** at 298K**.** Simulated spectra (black) obtained from single crystal structure (Mercury software).

Hirshfeld Surface Analysis

The effect of fluorination substitution at the axial position of synthesized porphyrin complexes (**1-5**) have also been assessed *via* Hirshfeld surfaces using Crystal Explorer.^[57]^ This method is particularly insightful as it quantifies the contributions of various intermolecular interactions within the crystal structure. The Hirshfeld surface representations are color-coded, where red spots highlight regions of close intermolecular interactions, such as hydrogen and halogen bonding, and blue spots indicate areas devoid of such close contacts (Figure S16-S20). The calculations revealed that halogen bond interactions involving iodine (I···I, I···F, I···π) are significant, contributing approximately 5-6% to the overall crystal structure in all the complexes. Interestingly, the study shows that increasing the fluorine content at the axial position does not significantly alter the halogen bond interactions involving the equatorial iodine atom. This suggests that the axial fluorination does not interfere with the established halogen bonding network, maintaining the structural integrity of the crystal.


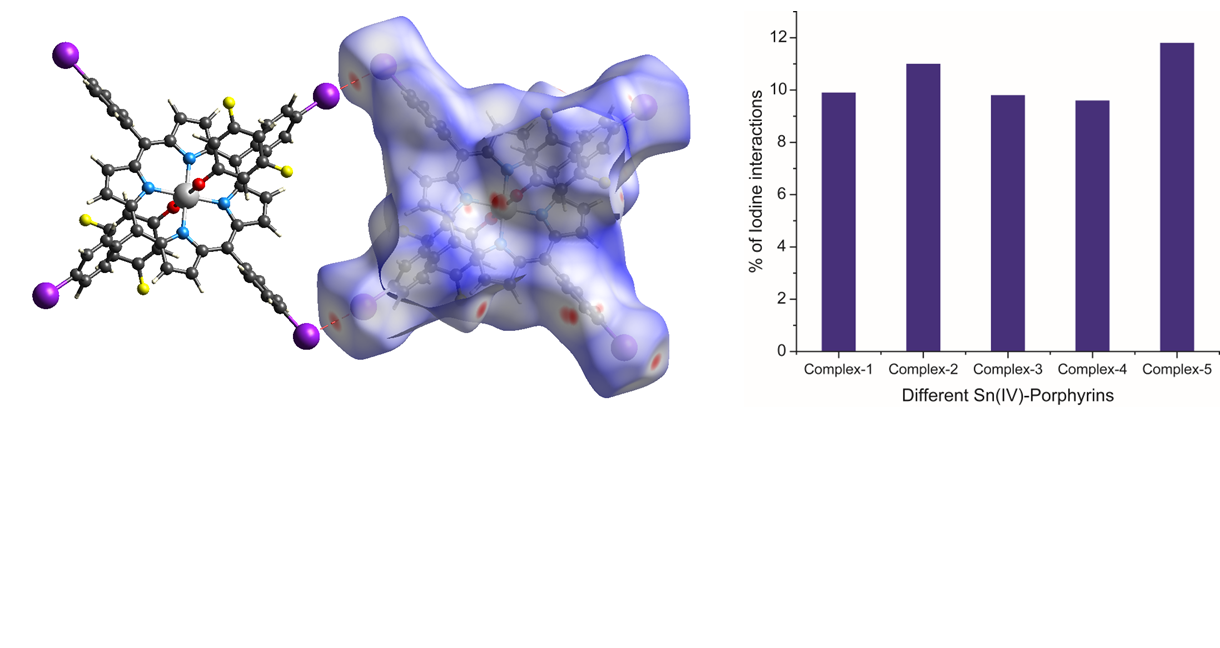


**Figure S16.** (A) Calculated Hirshfeld surfaces for the shortest intermolecular contacts represents the I···I in complex **3** (B) Contributions of halogen bond interactions involving iodine atom in all the five complexes.


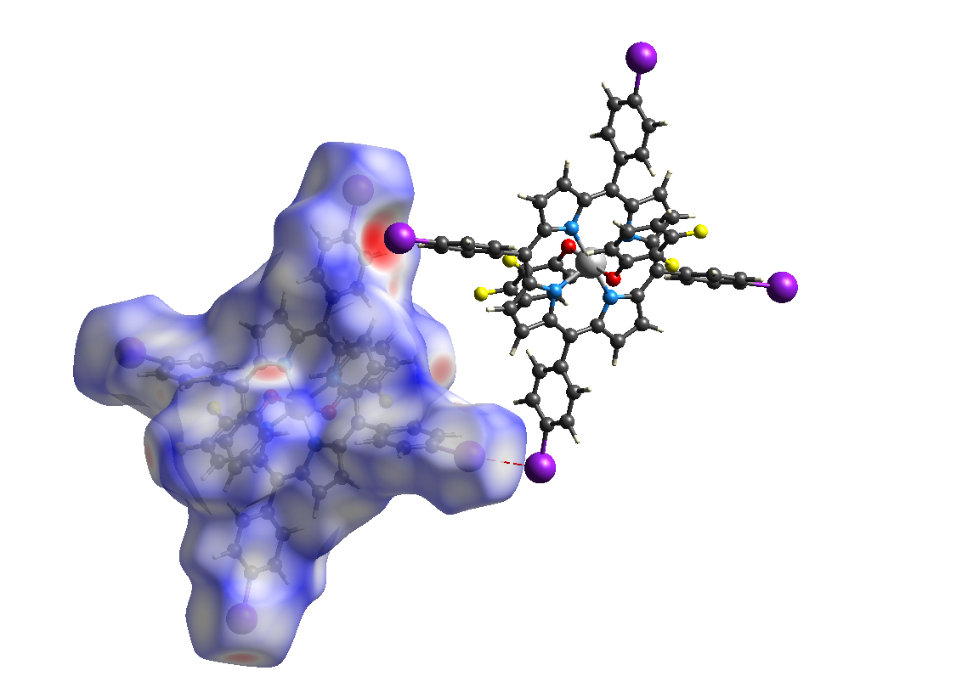


**Figure-S17:** Hirshfeld surface of Complex **1** indicating the short (I•••I) XB interaction

**
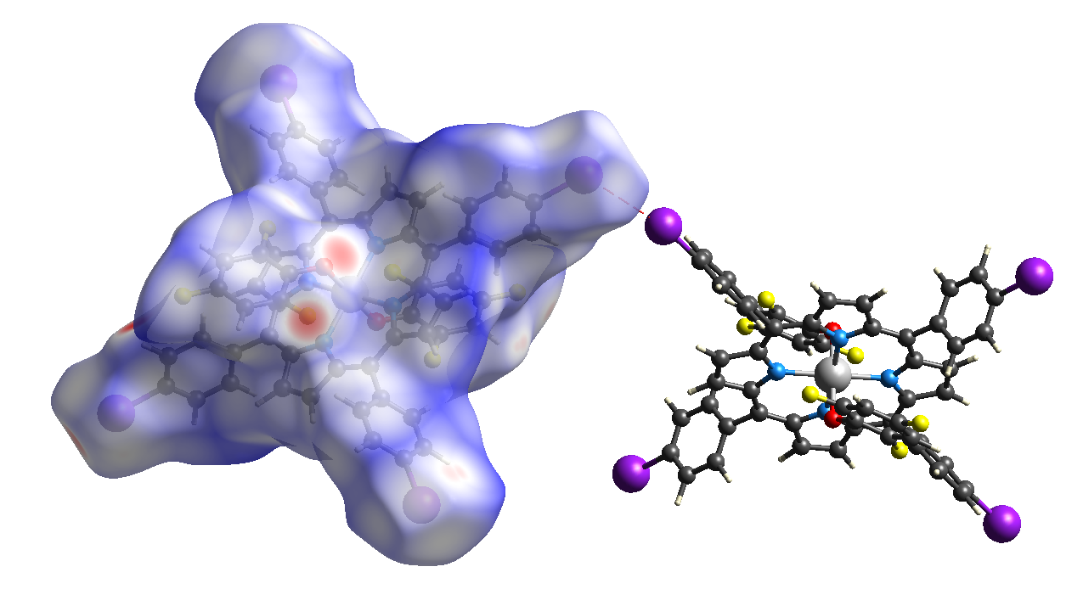
**

**Figure-S18:** Hirshfeld surface of Complex **2** indicating the short (I•••I) XB interaction

**
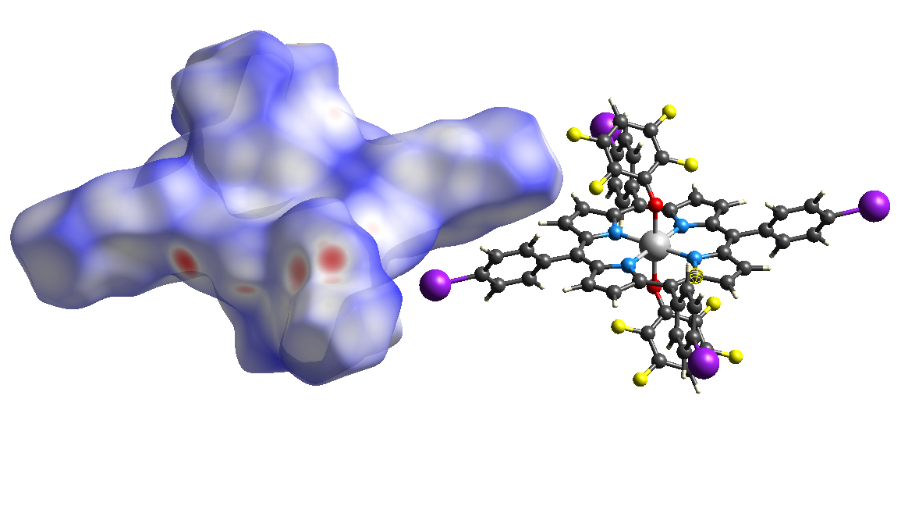
**

**Figure-S19:** Hirshfeld surface of Complex **4** indicating the short (I•••F) XB interaction

**
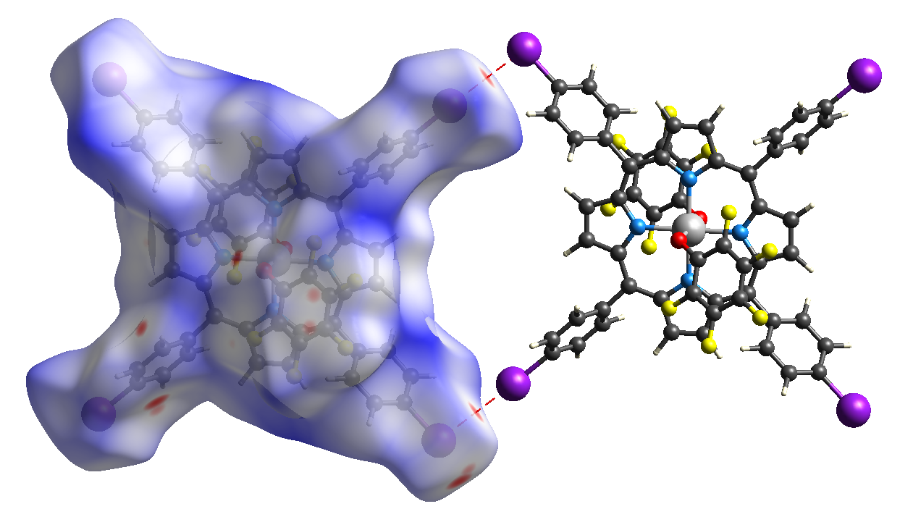
**

**Figure-S20:** Hirshfeld surface of Complex **5** indicating the short (I•••I) XB interaction.

**Electrochemical Study:**

Cyclic voltammetric studies of complex **1**-**5** were performed on a BAS Epsilon electrochemical workstation in dichloromethane with 0.1 M TBAP as the supporting electrolyte; the reference electrode was Ag/AgCl, and the auxiliary electrode was a platinum wire. The concentration of the complexes was on the order of 10^-3^ M. The ferrocene/ferrocenium couple occurs at E_1/2_ = 0.45 (65) V vs Ag/AgCl under the same experimental conditions.


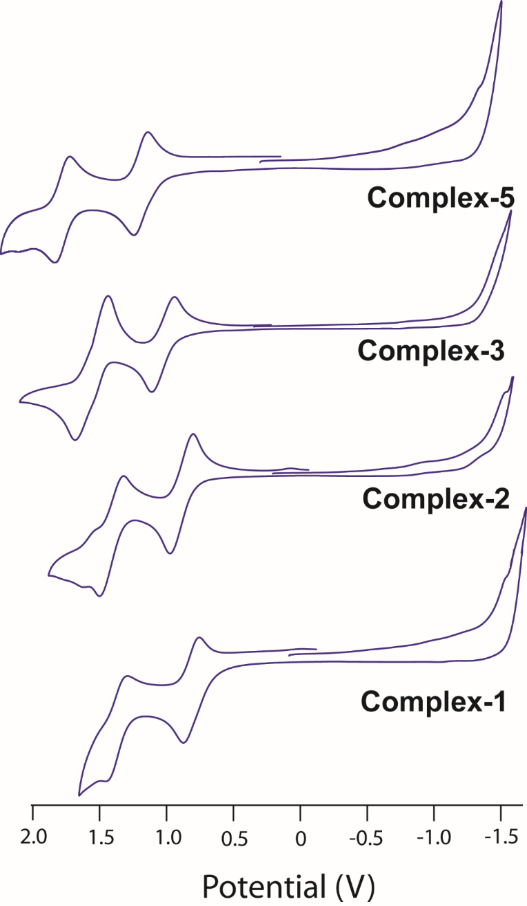


**Figure** **S21.** The cyclic voltammogram for complex 1, 2, 3 and 5 in CH_2_Cl_2_ scan rate100mV/s) with 0.1M tetra-n-butylammonium perchlorate as supporting electrolyte using a reference electrode of Ag/AgCl at 25 °C.

**Table-S3**: Electrochemical data for complexes **1**-**5**

| Complex | Oxidation (V)^a^ | | Reduction (V) ^b^ |
| --- | --- | --- | --- |
|  | E_1/2_(1)^a^ | E_1/2_(2)^a^ | Epc |
| 1 | 0.82 (72) | 1.35 (68) | -1.45^b^ |
| 2 | 0.86 (68) | 1.38(73) | -1.42 |
| 3 | 1.02 (73) | 1.42 (75) | -1.40 |
| 4 | 1.08 (75) | 1.52 (78) | -1.38 |
| 5 | 1.09 (76) | 1.54 (77) | -1.35 |

^a^Half-wave potentials (E1/2 in V) (Epa + Epc)/2 (peak potential differences in mV in parentheses). ^b^ Irreversible reduction processes, Epc.

**NCI analysis**


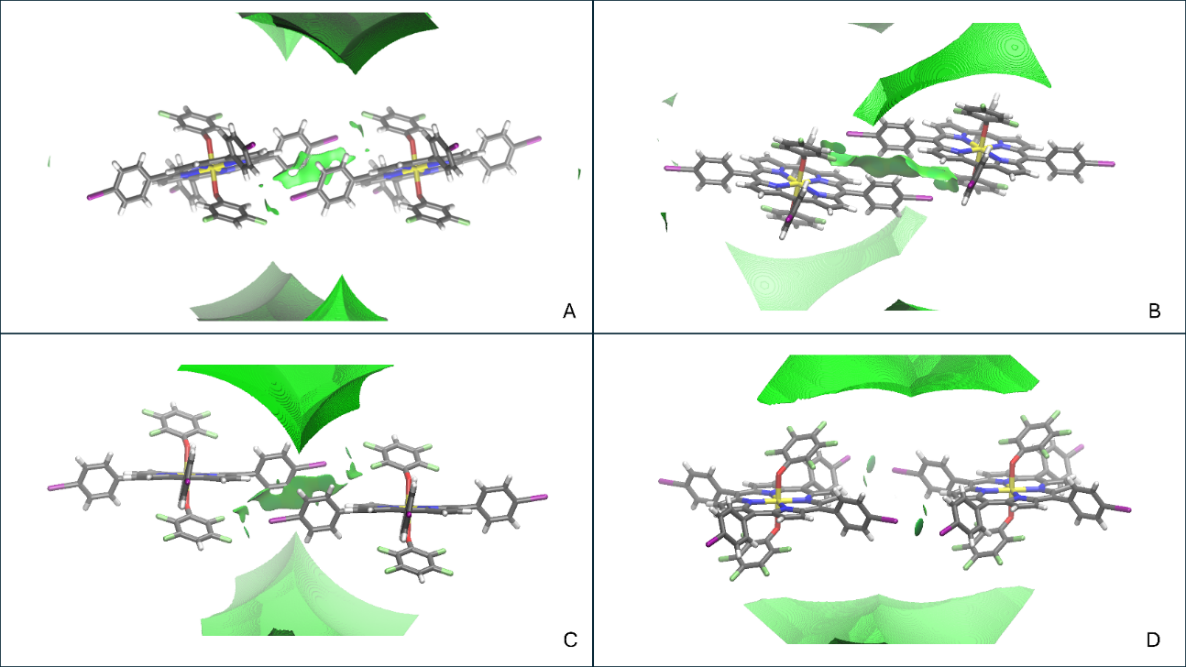


**Figure-S22:** Isofurface of the reduced gradient density (0.3 a.u.) of porphyrin dimers with 2 (A), 3 (B), 4 (C) and 5 (D) fluorine atoms in each aromatic ligand. Mapps obtained with the promolecular density, with grid spacing of 0.05 Å. Intramolecular interactions were removed.


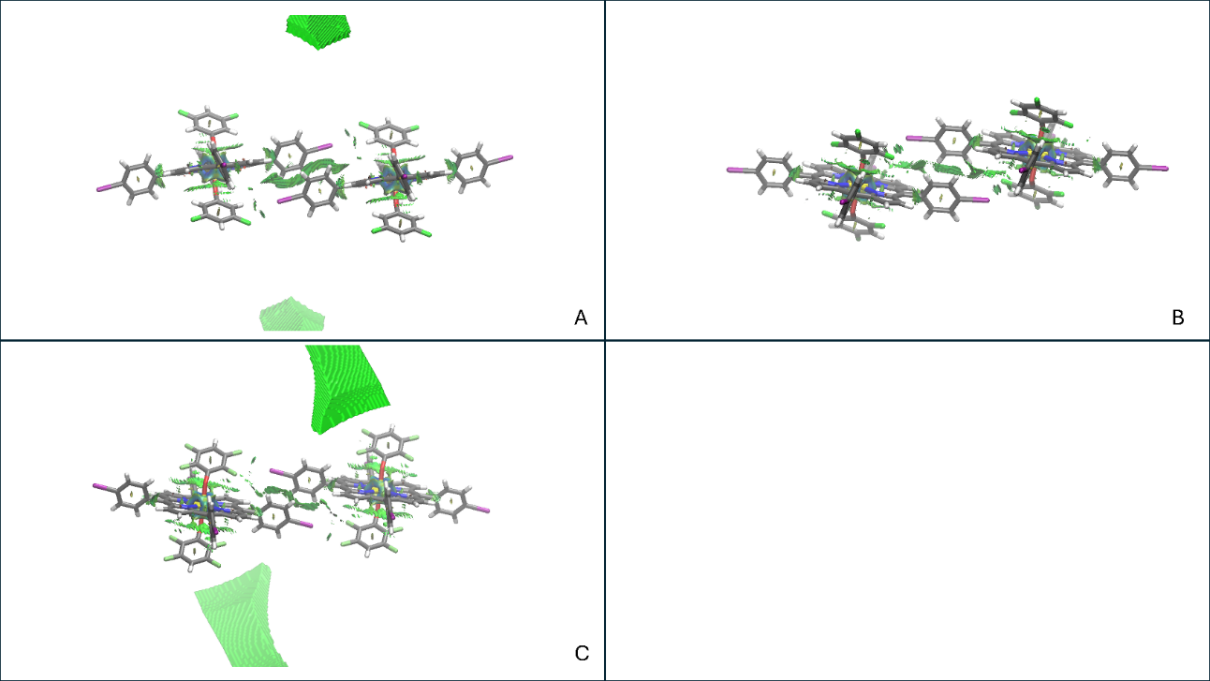


**Figure-S23:** Isofurface of the reduced gradient density (0.3 a.u.) of porphyrin dimers with 2 (A), 3 (B), 4 (C) fluorine atoms in each aromatic ligand. Mapps obtained with the electron density, with grid spacing of 1.5 Å. We highlight here the F…I interactions and the similarities with the maps obtained with promolecular density. Grid size of 1.5 Å was utilized due to high computational costs with lower values.

**Computational Details**

DFT calculations have been carried out by employing a B3LYP hybrid functional using, Gaussian 09, revision B.05, package.^58^ Using the method of Becke’s three-parameter hybrid exchange functional,^59^ the nonlocal correlation provided by the Lee, Yang, and Parr expression,^60^ and Vosko, Wilk, and Nussair 1980 correlation functional (III) for local correction. The basis set used during optimizations was Lanl2dz for the Sn and iodine-atom and 6-31G** for C, N, O, F and H-atom. The starting coordinates of the model complexes were derived from the experimental crystal structure of complex **5** ([Sn(IV)(L)₂] tetra-iodophenyl porphyrin, L = pentafluorophenolate). To generate the full series of fluorinated analogues (L = mono-, di-, tri-, and tetrafluorophenolate), the fluorine atoms were sequentially replaced with hydrogen atoms, while preserving the overall geometry of the parent structure. All model structures were subjected to full geometry optimization, which converged without imaginary frequencies, confirming that the obtained geometries correspond to true local minima. Input and output geometries for all model complexes are provided in **Table S4**. Resulting geometries were further used for single point calculations towards topological analysis of electron density and estimation of electric and electrostatic properties.

**Table-S4** Atomic coordinates (cartesian coordinates) before and after geometry optimization.

|  | Initial geometry | | |  | Optimized geometry | | |
| --- | --- | --- | --- | --- | --- | --- | --- |
| **Atom** | **X** | **Y** | **Z** |  | **X** | **Y** | **Z** |
| **1-F model** | | | | | | | |
| Sn | 16.5266 | 2.4738 | 12.3057 |  | 16.5266 | 2.47383 | 12.3057 |
| F | 19.1022 | 7.1286 | 16.5512 |  | 19.48622 | 7.71673 | 15.86461 |
| I | 8.7058 | 8.1988 | 10.7933 |  | 8.92187 | 8.68133 | 10.8668 |
| I | 15.8451 | 4.3599 | 21.9263 |  | 15.67824 | 3.68673 | 22.11613 |
| N | 15.5715 | 3.2783 | 10.6383 |  | 15.52038 | 3.22233 | 10.61845 |
| N | 15.2972 | 3.6148 | 13.5727 |  | 15.2357 | 3.55307 | 13.56369 |
| O | 17.9297 | 3.9947 | 12.2196 |  | 17.80551 | 4.01658 | 12.15227 |
| C | 15.8599 | 2.9973 | 9.3152 |  | 15.84606 | 2.96107 | 9.30887 |
| C | 15.0368 | 3.8127 | 8.4998 |  | 14.96338 | 3.73084 | 8.46566 |
| H | 15.0326 | 3.8301 | 7.5701 |  | 14.9893 | 3.73918 | 7.38596 |
| C | 14.257 | 4.5667 | 9.3208 |  | 14.12746 | 4.43711 | 9.28086 |
| H | 13.6323 | 5.1986 | 9.0489 |  | 13.35209 | 5.12752 | 8.98361 |
| C | 14.5656 | 4.2184 | 10.6769 |  | 14.47201 | 4.11273 | 10.64582 |
| C | 13.9452 | 4.7319 | 11.8233 |  | 13.84948 | 4.63579 | 11.80313 |
| C | 14.3159 | 4.4786 | 13.1669 |  | 14.20696 | 4.36396 | 13.14408 |
| C | 13.7452 | 5.0763 | 14.3258 |  | 13.54707 | 4.88925 | 14.31661 |
| H | 13.05 | 5.6938 | 14.3407 |  | 12.69119 | 5.54752 | 14.30491 |
| C | 14.4029 | 4.5776 | 15.4092 |  | 14.19997 | 4.40172 | 15.41076 |
| H | 14.2451 | 4.808 | 16.2962 |  | 13.97896 | 4.60007 | 16.44908 |
| C | 15.3757 | 3.6464 | 14.9529 |  | 15.26415 | 3.54889 | 14.93864 |
| C | 16.2489 | 2.8874 | 15.739 |  | 16.17559 | 2.84006 | 15.75099 |
| C | 12.7401 | 5.5829 | 11.603 |  | 12.7015 | 5.575 | 11.58696 |
| C | 12.699 | 6.9218 | 11.9734 |  | 12.82141 | 6.9377 | 11.89729 |
| H | 13.4389 | 7.3135 | 12.3798 |  | 13.75963 | 7.31631 | 12.29293 |
| C | 11.552 | 7.6778 | 11.7372 |  | 11.75803 | 7.81937 | 11.69639 |
| H | 11.521 | 8.5742 | 11.9843 |  | 11.87342 | 8.87033 | 11.93726 |
| C | 10.4667 | 7.088 | 11.1391 |  | 10.55764 | 7.33332 | 11.17968 |
| C | 10.4912 | 5.7511 | 10.7724 |  | 10.40971 | 5.98323 | 10.86405 |
| H | 9.7467 | 5.3557 | 10.3794 |  | 9.47364 | 5.60452 | 10.46835 |
| C | 11.6232 | 5.0238 | 10.9964 |  | 11.48245 | 5.11334 | 11.06896 |
| H | 11.6479 | 4.1314 | 10.7354 |  | 11.36613 | 4.05961 | 10.83177 |
| C | 16.2036 | 3.1378 | 17.2071 |  | 16.05275 | 3.02691 | 17.23352 |
| C | 17.1403 | 3.9314 | 17.8162 |  | 16.97974 | 3.82536 | 17.91919 |
| H | 17.8425 | 4.2747 | 17.3135 |  | 17.77807 | 4.3123 | 17.36566 |
| C | 17.0613 | 4.2352 | 19.1821 |  | 16.87952 | 4.01582 | 19.29873 |
| H | 17.7098 | 4.7728 | 19.5784 |  | 17.59997 | 4.64278 | 19.81234 |
| C | 16.0442 | 3.7474 | 19.9229 |  | 15.84304 | 3.39832 | 19.99787 |
| C | 15.116 | 2.926 | 19.3618 |  | 14.90967 | 2.59849 | 19.33957 |
| H | 14.4367 | 2.5628 | 19.8851 |  | 14.10707 | 2.11659 | 19.88701 |
| C | 15.1858 | 2.6302 | 17.9909 |  | 15.01957 | 2.41809 | 17.95899 |
| H | 14.5392 | 2.0849 | 17.6062 |  | 14.29807 | 1.79114 | 17.44245 |
| C | 18.2307 | 4.7507 | 13.2889 |  | 18.20078 | 4.90712 | 13.08153 |
| C | 17.5246 | 5.9174 | 13.5535 |  | 17.57017 | 6.16228 | 13.19074 |
| C | 17.8199 | 6.7258 | 14.6364 |  | 17.99833 | 7.10809 | 14.12211 |
| H | 17.3412 | 7.5073 | 14.7947 |  | 17.51622 | 8.07726 | 14.20742 |
| C | 18.8243 | 6.3459 | 15.4584 |  | 19.06775 | 6.80071 | 14.95522 |
| C | 19.5669 | 5.2336 | 15.2443 |  | 19.72279 | 5.57726 | 14.87127 |
| H | 20.2625 | 5.0087 | 15.8203 |  | 20.56729 | 5.3767 | 15.52403 |
| C | 19.267 | 4.4351 | 14.1466 |  | 19.28876 | 4.63735 | 13.93487 |
| I | 24.3474 | -3.2511 | 13.8181 |  | 24.13133 | -3.73367 | 13.74461 |
| I | 17.2081 | 0.5878 | 2.6851 |  | 17.37496 | 1.26092 | 2.49527 |
| N | 17.4817 | 1.6693 | 13.9731 |  | 17.53282 | 1.72532 | 13.99295 |
| N | 17.756 | 1.3329 | 11.0387 |  | 17.81751 | 1.39459 | 11.04771 |
| C | 17.1933 | 1.9504 | 15.2962 |  | 17.20714 | 1.98658 | 15.30254 |
| C | 18.0164 | 1.135 | 16.1116 |  | 18.08983 | 1.21682 | 16.14574 |
| H | 18.0206 | 1.1176 | 17.0413 |  | 18.06391 | 1.20848 | 17.22545 |
| C | 18.7962 | 0.381 | 15.2906 |  | 18.92575 | 0.51054 | 15.33054 |
| H | 19.4209 | -0.2509 | 15.5625 |  | 19.70112 | -0.17986 | 15.62779 |
| C | 18.4876 | 0.7293 | 13.9345 |  | 18.58119 | 0.83493 | 13.96558 |
| C | 19.108 | 0.2157 | 12.7881 |  | 19.20372 | 0.31186 | 12.80827 |
| C | 18.7373 | 0.469 | 11.4445 |  | 18.84625 | 0.5837 | 11.46732 |
| C | 19.308 | -0.1286 | 10.2856 |  | 19.50614 | 0.05841 | 10.29479 |
| H | 20.0032 | -0.7461 | 10.2707 |  | 20.36201 | -0.59987 | 10.30649 |
| C | 18.6503 | 0.3701 | 9.2022 |  | 18.85323 | 0.54593 | 9.20065 |
| H | 18.8081 | 0.1397 | 8.3152 |  | 19.07424 | 0.34758 | 8.16232 |
| C | 17.6775 | 1.3012 | 9.6585 |  | 17.78906 | 1.39877 | 9.67276 |
| C | 16.8043 | 2.0602 | 8.8724 |  | 16.87762 | 2.10759 | 8.86041 |
| C | 20.3131 | -0.6353 | 13.0084 |  | 20.3517 | -0.62734 | 13.02444 |
| C | 20.3542 | -1.9741 | 12.638 |  | 20.23179 | -1.99004 | 12.71412 |
| H | 19.6143 | -2.3659 | 12.2316 |  | 19.29357 | -2.36865 | 12.31848 |
| C | 21.5012 | -2.7301 | 12.8742 |  | 21.29518 | -2.87172 | 12.91501 |
| H | 21.5322 | -3.6266 | 12.6271 |  | 21.17979 | -3.92267 | 12.67415 |
| C | 22.5865 | -2.1404 | 13.4723 |  | 22.49557 | -2.38566 | 13.43172 |
| C | 22.562 | -0.8035 | 13.839 |  | 22.64349 | -1.03557 | 13.74734 |
| H | 23.3065 | -0.4081 | 14.232 |  | 23.57956 | -0.65686 | 14.14305 |
| C | 21.43 | -0.0762 | 13.615 |  | 21.57075 | -0.16569 | 13.54244 |
| H | 21.4053 | 0.8162 | 13.876 |  | 21.68707 | 0.88804 | 13.77962 |
| C | 16.8496 | 1.8099 | 7.4043 |  | 17.00045 | 1.92074 | 7.37788 |
| C | 15.913 | 1.0162 | 6.7952 |  | 16.07346 | 1.1223 | 6.69221 |
| H | 15.2107 | 0.673 | 7.2979 |  | 15.27512 | 0.63537 | 7.24574 |
| C | 15.9919 | 0.7125 | 5.4293 |  | 16.17367 | 0.93184 | 5.31267 |
| H | 15.3434 | 0.1748 | 5.033 |  | 15.45322 | 0.30489 | 4.79906 |
| C | 17.009 | 1.2003 | 4.6885 |  | 17.21016 | 1.54933 | 4.61353 |
| C | 17.9372 | 2.0216 | 5.2496 |  | 18.14354 | 2.34915 | 5.27183 |
| H | 18.6165 | 2.3849 | 4.7263 |  | 18.94615 | 2.83104 | 4.7244 |
| C | 17.8674 | 2.3175 | 6.6205 |  | 18.03364 | 2.52955 | 6.65241 |
| H | 18.514 | 2.8627 | 7.0052 |  | 18.75515 | 3.1565 | 7.16895 |
| F | 13.951 | -2.1809 | 8.0602 |  | 13.56697 | -2.76906 | 8.74678 |
| O | 15.1236 | 0.9529 | 12.3918 |  | 15.24769 | 0.93107 | 12.45913 |
| C | 14.8225 | 0.1969 | 11.3225 |  | 14.85242 | 0.04054 | 11.52987 |
| C | 15.5286 | -0.9697 | 11.0579 |  | 15.48303 | -1.21462 | 11.42065 |
| C | 15.2333 | -1.7782 | 9.975 |  | 15.05487 | -2.16042 | 10.48928 |
| H | 15.712 | -2.5596 | 9.8167 |  | 15.53698 | -3.1296 | 10.40396 |
| C | 14.2289 | -1.3982 | 9.153 |  | 13.98544 | -1.85304 | 9.65617 |
| C | 13.4863 | -0.286 | 9.3671 |  | 13.3304 | -0.62959 | 9.74013 |
| H | 12.7907 | -0.061 | 8.7911 |  | 12.48591 | -0.42903 | 9.08738 |
| C | 13.7862 | 0.5126 | 10.4648 |  | 13.76443 | 0.31032 | 10.67653 |
| H | 19.85449 | 3.56657 | 13.93351 |  | 19.7957 | 3.68176 | 13.84379 |
| H | 16.78371 | 6.21306 | 12.84036 |  | 16.7442 | 6.38587 | 12.52204 |
| H | 16.26949 | -1.26536 | 11.77104 |  | 16.309 | -1.43822 | 12.08934 |
| H | 13.19868 | 1.3811 | 10.67791 |  | 13.25751 | 1.2659 | 10.76762 |
| **2-F model** | | | | | | | |
| Sn | 2.857 | 3.9456 | 7.3563 |  | 2.85704 | 3.94561 | 7.3563 |
| F | 2.9086 | 8.5759 | 3.3692 |  | 2.37269 | 8.76688 | 3.64511 |
| F | 5.8013 | 5.046 | 2.2716 |  | 5.88387 | 5.91033 | 2.35264 |
| I | -4.9768 | 9.397 | 9.5455 |  | -4.73839 | 10.03253 | 9.25837 |
| I | 6.3141 | 5.3743 | 16.4341 |  | 6.34962 | 5.43882 | 16.52348 |
| N | 1.2054 | 4.6449 | 6.2867 |  | 1.16735 | 4.57577 | 6.28551 |
| N | 2.3455 | 5.1781 | 8.9614 |  | 2.29062 | 5.14999 | 8.98139 |
| O | 4.0581 | 5.4132 | 6.6148 |  | 3.97266 | 5.47972 | 6.66915 |
| C | 0.8808 | 4.2924 | 4.9949 |  | 0.84442 | 4.20847 | 4.99916 |
| C | -0.3186 | 4.9595 | 4.6624 |  | -0.38462 | 4.87253 | 4.63945 |
| H | -0.7693 | 4.8965 | 3.8515 |  | -0.88762 | 4.77261 | 3.68912 |
| C | -0.7012 | 5.7058 | 5.7247 |  | -0.76884 | 5.63425 | 5.70412 |
| H | -1.4558 | 6.2477 | 5.7703 |  | -1.63675 | 6.27282 | 5.7737 |
| C | 0.2575 | 5.5145 | 6.7634 |  | 0.20719 | 5.44595 | 6.75045 |
| C | 0.2136 | 6.0973 | 8.0287 |  | 0.18517 | 6.06635 | 8.02018 |
| C | 1.1982 | 5.924 | 9.0571 |  | 1.14474 | 5.91008 | 9.04615 |
| C | 1.0869 | 6.4174 | 10.4106 |  | 1.0709 | 6.49598 | 10.36363 |
| H | 0.3973 | 6.9399 | 10.7504 |  | 0.2729 | 7.13303 | 10.71503 |
| C | 2.1555 | 5.987 | 11.0801 |  | 2.16421 | 6.07631 | 11.0635 |
| H | 2.3454 | 6.175 | 11.9709 |  | 2.4163 | 6.30852 | 12.08757 |
| C | 2.9723 | 5.1786 | 10.1973 |  | 2.9381 | 5.2245 | 10.19326 |
| C | 4.1096 | 4.4931 | 10.5416 |  | 4.13111 | 4.55522 | 10.54219 |
| C | -0.9704 | 6.9141 | 8.3729 |  | -0.96508 | 6.98382 | 8.30756 |
| C | -0.8673 | 8.2436 | 8.7555 |  | -0.76475 | 8.3691 | 8.39625 |
| H | -0.0326 | 8.6528 | 8.7804 |  | 0.23091 | 8.77738 | 8.24725 |
| C | -2.0046 | 8.973 | 9.1012 |  | -1.8268 | 9.23478 | 8.66235 |
| H | -1.9335 | 9.8608 | 9.3686 |  | -1.65248 | 10.30349 | 8.71983 |
| C | -3.2144 | 8.3655 | 9.0409 |  | -3.10506 | 8.7089 | 8.84568 |
| C | -3.3599 | 7.0681 | 8.6481 |  | -3.33243 | 7.33538 | 8.76433 |
| H | -4.2021 | 6.6765 | 8.6022 |  | -4.32648 | 6.92743 | 8.91223 |
| C | -2.2247 | 6.3469 | 8.3214 |  | -2.26108 | 6.48126 | 8.49473 |
| H | -2.3104 | 5.4588 | 8.06 |  | -2.43437 | 5.41023 | 8.43875 |
| C | 4.6467 | 4.6836 | 11.9349 |  | 4.6476 | 4.76737 | 11.93308 |
| C | 4.572 | 3.687 | 12.8617 |  | 4.54207 | 3.74792 | 12.8908 |
| H | 4.1886 | 2.8742 | 12.6227 |  | 4.07049 | 2.80645 | 12.6227 |
| C | 5.0499 | 3.8541 | 14.1432 |  | 5.01941 | 3.93265 | 14.18972 |
| H | 4.9959 | 3.1646 | 14.7653 |  | 4.92467 | 3.13577 | 14.91903 |
| C | 5.6045 | 5.0571 | 14.4787 |  | 5.60871 | 5.14887 | 14.53323 |
| C | 5.7011 | 6.0658 | 13.5562 |  | 5.72297 | 6.17905 | 13.60052 |
| H | 6.1054 | 6.8731 | 13.7792 |  | 6.18355 | 7.12334 | 13.86969 |
| C | 5.1878 | 5.8643 | 12.2909 |  | 5.24019 | 5.98228 | 12.305 |
| H | 5.217 | 6.557 | 11.6716 |  | 5.33433 | 6.77972 | 11.57312 |
| C | 4.1401 | 5.8441 | 5.3539 |  | 4.00461 | 6.06865 | 5.46849 |
| C | 3.45 | 6.9865 | 4.9699 |  | 3.13244 | 7.13441 | 5.16408 |
| H | 2.9172 | 7.4443 | 5.5793 |  | 2.41078 | 7.47778 | 5.89559 |
| C | 3.565 | 7.4195 | 3.709 |  | 3.2154 | 7.7457 | 3.9216 |
| C | 4.3429 | 6.8016 | 2.7498 |  | 4.1247 | 7.35989 | 2.94048 |
| H | 4.4073 | 7.1208 | 1.8787 |  | 4.17926 | 7.86018 | 1.98169 |
| C | 5.0058 | 5.6967 | 3.1676 |  | 4.97861 | 6.31179 | 3.27314 |
| C | 4.9412 | 5.2152 | 4.4123 |  | 4.94322 | 5.66127 | 4.49773 |
| H | 5.4325 | 4.4608 | 4.646 |  | 5.6342 | 4.8539 | 4.70928 |
| I | 10.6909 | -1.5058 | 5.1671 |  | 10.45246 | -2.14133 | 5.45423 |
| I | -0.6 | 2.5169 | -1.7215 |  | -0.63555 | 2.45239 | -1.81088 |
| N | 4.5087 | 3.2463 | 8.4259 |  | 4.54672 | 3.31544 | 8.42709 |
| N | 3.3686 | 2.7131 | 5.7512 |  | 3.42345 | 2.74122 | 5.7312 |
| C | 4.8333 | 3.5988 | 9.7177 |  | 4.86965 | 3.68274 | 9.71343 |
| C | 6.0327 | 2.9317 | 10.0502 |  | 6.0987 | 3.01868 | 10.07315 |
| H | 6.4834 | 2.9947 | 10.8611 |  | 6.60169 | 3.1186 | 11.02347 |
| C | 6.4152 | 2.1854 | 8.9879 |  | 6.48291 | 2.25696 | 9.00848 |
| H | 7.1699 | 1.6435 | 8.9423 |  | 7.35082 | 1.61839 | 8.9389 |
| C | 5.4565 | 2.3768 | 7.9492 |  | 5.50688 | 2.44526 | 7.96215 |
| C | 5.5005 | 1.794 | 6.6839 |  | 5.5289 | 1.82486 | 6.69242 |
| C | 4.5158 | 1.9672 | 5.6555 |  | 4.56933 | 1.98113 | 5.66645 |
| C | 4.6272 | 1.4738 | 4.302 |  | 4.64318 | 1.39523 | 4.34896 |
| H | 5.3168 | 0.9513 | 3.9622 |  | 5.44118 | 0.75818 | 3.99756 |
| C | 3.5586 | 1.9042 | 3.6325 |  | 3.54987 | 1.8149 | 3.64909 |
| H | 3.3687 | 1.7163 | 2.7417 |  | 3.29778 | 1.5827 | 2.62502 |
| C | 2.7418 | 2.7126 | 4.5153 |  | 2.77597 | 2.66671 | 4.51933 |
| C | 1.6045 | 3.3981 | 4.171 |  | 1.58296 | 3.33599 | 4.1704 |
| C | 6.6845 | 0.9771 | 6.3397 |  | 6.67915 | 0.90739 | 6.40504 |
| C | 6.5814 | -0.3524 | 5.9571 |  | 6.47883 | -0.47789 | 6.31634 |
| H | 5.7466 | -0.7615 | 5.9322 |  | 5.48317 | -0.88617 | 6.46534 |
| C | 7.7187 | -1.0817 | 5.6114 |  | 7.54087 | -1.34357 | 6.05024 |
| H | 7.6476 | -1.9696 | 5.344 |  | 7.36655 | -2.41228 | 5.99276 |
| C | 8.9285 | -0.4743 | 5.6717 |  | 8.81913 | -0.81769 | 5.86691 |
| C | 9.0739 | 0.8231 | 6.0645 |  | 9.0465 | 0.55583 | 5.94827 |
| H | 9.9162 | 1.2147 | 6.1104 |  | 10.04055 | 0.96378 | 5.80038 |
| C | 7.9388 | 1.5443 | 6.3911 |  | 7.97516 | 1.40995 | 6.21787 |
| H | 8.0244 | 2.4324 | 6.6526 |  | 8.14844 | 2.48098 | 6.27386 |
| C | 1.0674 | 3.2076 | 2.7777 |  | 1.06647 | 3.12384 | 2.77952 |
| C | 1.1421 | 4.2043 | 1.8508 |  | 1.172 | 4.14329 | 1.8218 |
| H | 1.5254 | 5.017 | 2.0899 |  | 1.64357 | 5.08477 | 2.0899 |
| C | 0.6642 | 4.0371 | 0.5694 |  | 0.69466 | 3.95856 | 0.52287 |
| H | 0.7181 | 4.7266 | -0.0527 |  | 0.78939 | 4.75545 | -0.20644 |
| C | 0.1096 | 2.8341 | 0.2339 |  | 0.10536 | 2.74234 | 0.17937 |
| C | 0.0129 | 1.8254 | 1.1564 |  | -0.00889 | 1.71216 | 1.11208 |
| H | -0.3914 | 1.0181 | 0.9334 |  | -0.46947 | 0.76787 | 0.8429 |
| C | 0.5263 | 2.0269 | 2.4217 |  | 0.47389 | 1.90893 | 2.40759 |
| H | 0.4971 | 1.3342 | 3.041 |  | 0.37976 | 1.11149 | 3.13947 |
| F | 2.8055 | -0.6847 | 11.3434 |  | 3.34139 | -0.87567 | 11.06748 |
| F | -0.0872 | 2.8452 | 12.441 |  | -0.16979 | 1.98088 | 12.35995 |
| O | 1.656 | 2.478 | 8.0978 |  | 1.74142 | 2.4115 | 8.04345 |
| C | 1.5739 | 2.0471 | 9.3587 |  | 1.70947 | 1.82256 | 9.2441 |
| C | 2.2641 | 0.9047 | 9.7427 |  | 2.58164 | 0.75681 | 9.54851 |
| H | 2.7969 | 0.4469 | 9.1333 |  | 3.3033 | 0.41344 | 8.817 |
| C | 2.1491 | 0.4717 | 11.0035 |  | 2.49868 | 0.14551 | 10.791 |
| C | 1.3712 | 1.0896 | 11.9628 |  | 1.58938 | 0.53132 | 11.77212 |
| H | 1.3068 | 0.7704 | 12.8339 |  | 1.53482 | 0.03103 | 12.7309 |
| C | 0.7082 | 2.1945 | 11.545 |  | 0.73547 | 1.57942 | 11.43945 |
| C | 0.7729 | 2.676 | 10.3003 |  | 0.77086 | 2.22994 | 10.21486 |
| H | 0.2816 | 3.4304 | 10.0666 |  | 0.07987 | 3.03731 | 10.00332 |
| **3-F model** | | | | | | | |
| Sn | 16.5266 | 2.4738 | 12.3057 |  | 16.5266 | 2.47383 | 12.3057 |
| F | 16.622 | 6.2776 | 12.6847 |  | 16.40737 | 6.40039 | 12.64681 |
| F | 19.1022 | 7.1286 | 16.5512 |  | 19.64661 | 7.64462 | 15.84283 |
| F | 19.9725 | 3.3921 | 13.8907 |  | 20.08101 | 3.56109 | 13.5225 |
| I | 8.7058 | 8.1988 | 10.7933 |  | 8.96711 | 8.69135 | 10.8311 |
| I | 15.8451 | 4.3599 | 21.9263 |  | 15.57488 | 3.65184 | 22.12246 |
| N | 15.5715 | 3.2783 | 10.6383 |  | 15.50157 | 3.17064 | 10.61374 |
| N | 15.2972 | 3.6148 | 13.5727 |  | 15.22603 | 3.53625 | 13.56127 |
| O | 17.9297 | 3.9947 | 12.2196 |  | 17.76582 | 4.0637 | 12.10455 |
| C | 15.8599 | 2.9973 | 9.3152 |  | 15.83477 | 2.92 | 9.30226 |
| C | 15.0368 | 3.8127 | 8.4998 |  | 14.94981 | 3.68786 | 8.46094 |
| H | 15.0326 | 3.8301 | 7.5701 |  | 14.98044 | 3.70365 | 7.38155 |
| C | 14.257 | 4.5667 | 9.3208 |  | 14.10851 | 4.38613 | 9.27706 |
| H | 13.6323 | 5.1986 | 9.0489 |  | 13.32983 | 5.07348 | 8.98178 |
| C | 14.5656 | 4.2184 | 10.6769 |  | 14.45197 | 4.06005 | 10.63947 |
| C | 13.9452 | 4.7319 | 11.8233 |  | 13.82982 | 4.58553 | 11.79116 |
| C | 14.3159 | 4.4786 | 13.1669 |  | 14.18591 | 4.32771 | 13.13169 |
| C | 13.7452 | 5.0763 | 14.3258 |  | 13.51714 | 4.85664 | 14.29485 |
| H | 13.05 | 5.6938 | 14.3407 |  | 12.65466 | 5.5059 | 14.27218 |
| C | 14.4029 | 4.5776 | 15.4092 |  | 14.17235 | 4.38882 | 15.39655 |
| H | 14.2451 | 4.808 | 16.2962 |  | 13.94613 | 4.59716 | 16.43171 |
| C | 15.3757 | 3.6464 | 14.9529 |  | 15.24873 | 3.54535 | 14.93781 |
| C | 16.2489 | 2.8874 | 15.739 |  | 16.16979 | 2.85904 | 15.75798 |
| C | 12.7401 | 5.5829 | 11.603 |  | 12.69329 | 5.53451 | 11.56949 |
| C | 12.699 | 6.9218 | 11.9734 |  | 12.85716 | 6.90388 | 11.82301 |
| H | 13.4389 | 7.3135 | 12.3798 |  | 13.81884 | 7.26729 | 12.17458 |
| C | 11.552 | 7.6778 | 11.7372 |  | 11.8069 | 7.79971 | 11.61556 |
| H | 11.521 | 8.5742 | 11.9843 |  | 11.94988 | 8.85694 | 11.80959 |
| C | 10.4667 | 7.088 | 11.1391 |  | 10.58258 | 7.3185 | 11.15241 |
| C | 10.4912 | 5.7511 | 10.7724 |  | 10.39468 | 5.96095 | 10.89411 |
| H | 9.7467 | 5.3557 | 10.3794 |  | 9.43935 | 5.59012 | 10.53887 |
| C | 11.6232 | 5.0238 | 10.9964 |  | 11.45394 | 5.07504 | 11.10312 |
| H | 11.6479 | 4.1314 | 10.7354 |  | 11.31747 | 4.01424 | 10.91115 |
| C | 16.2036 | 3.1378 | 17.2071 |  | 16.0286 | 3.03354 | 17.2391 |
| C | 17.1403 | 3.9314 | 17.8162 |  | 16.97822 | 3.77417 | 17.95842 |
| H | 17.8425 | 4.2747 | 17.3135 |  | 17.81346 | 4.22761 | 17.43176 |
| C | 17.0613 | 4.2352 | 19.1821 |  | 16.85687 | 3.95189 | 19.3378 |
| H | 17.7098 | 4.7728 | 19.5784 |  | 17.59646 | 4.53471 | 19.87565 |
| C | 16.0442 | 3.7474 | 19.9229 |  | 15.77454 | 3.37995 | 20.0054 |
| C | 15.116 | 2.926 | 19.3618 |  | 14.81804 | 2.63647 | 19.31499 |
| H | 14.4367 | 2.5628 | 19.8851 |  | 13.98048 | 2.1876 | 19.83759 |
| C | 15.1858 | 2.6302 | 17.9909 |  | 14.95056 | 2.46823 | 17.93518 |
| H | 14.5392 | 2.0849 | 17.6062 |  | 14.2116 | 1.88337 | 17.39475 |
| C | 18.2307 | 4.7507 | 13.2889 |  | 18.20267 | 4.91174 | 13.03391 |
| C | 17.5246 | 5.9174 | 13.5535 |  | 17.54881 | 6.12618 | 13.32026 |
| C | 17.8199 | 6.7258 | 14.6364 |  | 18.0046 | 7.05233 | 14.25085 |
| H | 17.3412 | 7.5073 | 14.7947 |  | 17.46028 | 7.97168 | 14.43103 |
| C | 18.8243 | 6.3459 | 15.4584 |  | 19.18038 | 6.76186 | 14.93218 |
| C | 19.5669 | 5.2336 | 15.2443 |  | 19.89205 | 5.58999 | 14.70285 |
| H | 20.2625 | 5.0087 | 15.8203 |  | 20.81555 | 5.37842 | 15.22877 |
| C | 19.267 | 4.4351 | 14.1466 |  | 19.39123 | 4.69952 | 13.75945 |
| I | 24.3474 | -3.2511 | 13.8181 |  | 24.08609 | -3.74371 | 13.7803 |
| I | 17.2081 | 0.5878 | 2.6851 |  | 17.47833 | 1.29581 | 2.48894 |
| N | 17.4817 | 1.6693 | 13.9731 |  | 17.55163 | 1.77701 | 13.99766 |
| N | 17.756 | 1.3329 | 11.0387 |  | 17.82717 | 1.4114 | 11.05013 |
| C | 17.1933 | 1.9504 | 15.2962 |  | 17.21843 | 2.02766 | 15.30914 |
| C | 18.0164 | 1.135 | 16.1116 |  | 18.10339 | 1.25979 | 16.15046 |
| H | 18.0206 | 1.1176 | 17.0413 |  | 18.07276 | 1.24401 | 17.22985 |
| C | 18.7962 | 0.381 | 15.2906 |  | 18.94469 | 0.56153 | 15.33434 |
| H | 19.4209 | -0.2509 | 15.5625 |  | 19.72337 | -0.12583 | 15.62962 |
| C | 18.4876 | 0.7293 | 13.9345 |  | 18.60123 | 0.8876 | 13.97193 |
| C | 19.108 | 0.2157 | 12.7881 |  | 19.22339 | 0.36212 | 12.82024 |
| C | 18.7373 | 0.469 | 11.4445 |  | 18.86729 | 0.61994 | 11.47971 |
| C | 19.308 | -0.1286 | 10.2856 |  | 19.53606 | 0.09102 | 10.31655 |
| H | 20.0032 | -0.7461 | 10.2707 |  | 20.39855 | -0.55824 | 10.33921 |
| C | 18.6503 | 0.3701 | 9.2022 |  | 18.88085 | 0.55884 | 9.21485 |
| H | 18.8081 | 0.1397 | 8.3152 |  | 19.10707 | 0.3505 | 8.17969 |
| C | 17.6775 | 1.3012 | 9.6585 |  | 17.80448 | 1.4023 | 9.6736 |
| C | 16.8043 | 2.0602 | 8.8724 |  | 16.88341 | 2.08862 | 8.85342 |
| C | 20.3131 | -0.6353 | 13.0084 |  | 20.35991 | -0.58686 | 13.04191 |
| C | 20.3542 | -1.9741 | 12.638 |  | 20.19604 | -1.95623 | 12.78839 |
| H | 19.6143 | -2.3659 | 12.2316 |  | 19.23436 | -2.31964 | 12.43682 |
| C | 21.5012 | -2.7301 | 12.8742 |  | 21.2463 | -2.85206 | 12.99584 |
| H | 21.5322 | -3.6266 | 12.6271 |  | 21.10332 | -3.90929 | 12.80181 |
| C | 22.5865 | -2.1404 | 13.4723 |  | 22.47062 | -2.37086 | 13.45899 |
| C | 22.562 | -0.8035 | 13.839 |  | 22.65852 | -1.0133 | 13.71729 |
| H | 23.3065 | -0.4081 | 14.232 |  | 23.61385 | -0.64248 | 14.07253 |
| C | 21.43 | -0.0762 | 13.615 |  | 21.59927 | -0.12739 | 13.50828 |
| H | 21.4053 | 0.8162 | 13.876 |  | 21.73573 | 0.93341 | 13.70025 |
| C | 16.8496 | 1.8099 | 7.4043 |  | 17.0246 | 1.91412 | 7.3723 |
| C | 15.913 | 1.0162 | 6.7952 |  | 16.07498 | 1.17348 | 6.65298 |
| H | 15.2107 | 0.673 | 7.2979 |  | 15.23974 | 0.72005 | 7.17964 |
| C | 15.9919 | 0.7125 | 5.4293 |  | 16.19634 | 0.99577 | 5.2736 |
| H | 15.3434 | 0.1748 | 5.033 |  | 15.45674 | 0.41294 | 4.73575 |
| C | 17.009 | 1.2003 | 4.6885 |  | 17.27866 | 1.5677 | 4.606 |
| C | 17.9372 | 2.0216 | 5.2496 |  | 18.23516 | 2.31119 | 5.29641 |
| H | 18.6165 | 2.3849 | 4.7263 |  | 19.07273 | 2.76006 | 4.77381 |
| C | 17.8674 | 2.3175 | 6.6205 |  | 18.10265 | 2.47942 | 6.67622 |
| H | 18.514 | 2.8627 | 7.0052 |  | 18.84161 | 3.06428 | 7.21666 |
| F | 16.4312 | -1.3299 | 11.9267 |  | 16.64582 | -1.45274 | 11.9646 |
| F | 13.951 | -2.1809 | 8.0602 |  | 13.4066 | -2.69697 | 8.76857 |
| F | 13.0807 | 1.5555 | 10.7207 |  | 12.97221 | 1.38658 | 11.08888 |
| O | 15.1236 | 0.9529 | 12.3918 |  | 15.28738 | 0.88396 | 12.50685 |
| C | 14.8225 | 0.1969 | 11.3225 |  | 14.85054 | 0.03592 | 11.57749 |
| C | 15.5286 | -0.9697 | 11.0579 |  | 15.50439 | -1.17852 | 11.29115 |
| C | 15.2333 | -1.7782 | 9.975 |  | 15.04861 | -2.10467 | 10.36056 |
| H | 15.712 | -2.5596 | 9.8167 |  | 15.59292 | -3.02403 | 10.18038 |
| C | 14.2289 | -1.3982 | 9.153 |  | 13.87283 | -1.81421 | 9.67921 |
| C | 13.4863 | -0.286 | 9.3671 |  | 13.16116 | -0.64233 | 9.90854 |
| H | 12.7907 | -0.061 | 8.7911 |  | 12.23767 | -0.43076 | 9.38262 |
| C | 13.7862 | 0.5126 | 10.4648 |  | 13.66198 | 0.24815 | 10.85194 |
| **4-F model** | | | | | | | |
| Sn | 2.3325 | 4.6968 | 7.4277 |  | 2.33249 | 4.6969 | 7.42769 |
| F | 2.2933 | 0.8856 | 8.423 |  | 1.82708 | 0.77514 | 7.34 |
| F | 2.5554 | -0.3353 | 10.7746 |  | 2.38726 | -1.20341 | 9.04719 |
| F | 0.2146 | 3.1815 | 12.841 |  | 1.55607 | 1.69525 | 12.70782 |
| F | -0.0266 | 4.4064 | 10.5117 |  | 0.99731 | 3.6882 | 11.0145 |
| I | -1.7106 | 3.8892 | -1.4787 |  | -1.48428 | 3.35458 | -1.61635 |
| I | -4.7339 | 11.1225 | 9.6931 |  | -4.12404 | 12.04592 | 9.00512 |
| N | 2.6649 | 3.4241 | 5.7995 |  | 2.72459 | 3.39603 | 5.83229 |
| N | 0.7361 | 5.5703 | 6.4235 |  | 0.66488 | 5.44087 | 6.41297 |
| O | 1.0068 | 3.3151 | 8.2091 |  | 1.1006 | 3.29716 | 8.26504 |
| C | 3.68 | 2.5117 | 5.6807 |  | 3.74211 | 2.47266 | 5.77972 |
| C | 3.6024 | 1.9217 | 4.369 |  | 3.6615 | 1.78501 | 4.51403 |
| H | 4.1674 | 1.2676 | 4.0254 |  | 4.33373 | 1.0039 | 4.19106 |
| C | 2.5653 | 2.4879 | 3.7361 |  | 2.60277 | 2.30585 | 3.83044 |
| H | 2.2785 | 2.2842 | 2.8755 |  | 2.25089 | 2.0268 | 2.84824 |
| C | 1.9715 | 3.4566 | 4.6052 |  | 2.00713 | 3.32317 | 4.66166 |
| C | 0.9134 | 4.3195 | 4.2932 |  | 0.88589 | 4.10671 | 4.32375 |
| C | 0.3586 | 5.3025 | 5.1206 |  | 0.27282 | 5.07843 | 5.14082 |
| C | -0.6798 | 6.2361 | 4.7805 |  | -0.84901 | 5.9018 | 4.76779 |
| H | -1.098 | 6.3042 | 3.9533 |  | -1.36777 | 5.84641 | 3.82208 |
| C | -0.9451 | 6.9897 | 5.856 |  | -1.10451 | 6.74949 | 5.80697 |
| H | -1.5899 | 7.6574 | 5.9112 |  | -1.86945 | 7.51001 | 5.8614 |
| C | -0.0577 | 6.5832 | 6.9077 |  | -0.14635 | 6.46908 | 6.84597 |
| C | 0.016 | 7.1628 | 8.1809 |  | -0.03416 | 7.16127 | 8.0697 |
| C | 0.3026 | 4.1925 | 2.9206 |  | 0.32476 | 3.92664 | 2.94422 |
| C | -0.8749 | 3.5484 | 2.7512 |  | -0.82757 | 3.16062 | 2.7253 |
| H | -1.2976 | 3.1626 | 3.484 |  | -1.32288 | 2.68258 | 3.56597 |
| C | -1.4606 | 3.4591 | 1.4945 |  | -1.34456 | 2.99527 | 1.4383 |
| H | -2.2659 | 3.0055 | 1.387 |  | -2.23468 | 2.39521 | 1.2842 |
| C | -0.8568 | 4.0319 | 0.4293 |  | -0.70017 | 3.60649 | 0.36365 |
| C | 0.2818 | 4.6833 | 0.5942 |  | 0.44527 | 4.37799 | 0.55702 |
| H | 0.6847 | 5.0973 | -0.1352 |  | 0.94004 | 4.85723 | -0.28054 |
| C | 0.8852 | 4.7587 | 1.845 |  | 0.95466 | 4.53425 | 1.84787 |
| H | 1.6968 | 5.2024 | 1.9407 |  | 1.84105 | 5.14242 | 2.00532 |
| C | -1.0679 | 8.1385 | 8.53 |  | -0.9907 | 8.29338 | 8.29837 |
| C | -0.808 | 9.4816 | 8.7974 |  | -0.56542 | 9.61794 | 8.11634 |
| H | 0.064 | 9.802 | 8.7502 |  | 0.45584 | 9.81722 | 7.80368 |
| C | -1.8431 | 10.344 | 9.1316 |  | -1.44673 | 10.68314 | 8.31426 |
| H | -1.6685 | 11.2359 | 9.3264 |  | -1.10574 | 11.70165 | 8.16347 |
| C | -3.1341 | 9.8586 | 9.1732 |  | -2.76073 | 10.41934 | 8.70004 |
| C | -3.4215 | 8.5466 | 8.8909 |  | -3.20512 | 9.11144 | 8.88841 |
| H | -4.2977 | 8.2343 | 8.9165 |  | -4.22643 | 8.91003 | 9.19195 |
| C | -2.3699 | 7.693 | 8.5686 |  | -2.31597 | 8.05411 | 8.68459 |
| H | -2.5508 | 6.8017 | 8.3725 |  | -2.65614 | 7.03305 | 8.83382 |
| C | 1.1418 | 2.702 | 9.3767 |  | 1.37937 | 2.3124 | 9.10649 |
| C | 1.7866 | 1.4732 | 9.4985 |  | 1.72623 | 1.01895 | 8.6641 |
| C | 1.8896 | 0.853 | 10.733 |  | 2.01934 | -0.0078 | 9.55314 |
| C | 1.4071 | 1.3662 | 11.865 |  | 1.96002 | 0.17731 | 10.92926 |
| H | 1.509 | 0.9383 | 12.6836 |  | 2.19376 | -0.62491 | 11.6177 |
| C | 0.7405 | 2.5816 | 11.7461 |  | 1.60433 | 1.44179 | 11.38356 |
| C | 0.6271 | 3.2335 | 10.5518 |  | 1.3101 | 2.47743 | 10.50533 |
| I | 6.3755 | 5.5044 | 16.3341 |  | 6.14889 | 6.03885 | 16.47192 |
| I | 9.3989 | -1.7289 | 5.1622 |  | 8.78848 | -2.65263 | 5.85063 |
| N | 2 | 5.9695 | 9.0558 |  | 1.94035 | 5.99775 | 9.02311 |
| N | 3.9289 | 3.8233 | 8.4319 |  | 4.00007 | 3.95291 | 8.44244 |
| C | 0.9849 | 6.8819 | 9.1747 |  | 0.9228 | 6.92107 | 9.07571 |
| C | 1.0625 | 7.4719 | 10.4864 |  | 1.0034 | 7.60871 | 10.34141 |
| H | 0.4975 | 8.126 | 10.83 |  | 0.33114 | 8.3898 | 10.66439 |
| C | 2.0997 | 6.9057 | 11.1192 |  | 2.06213 | 7.08787 | 11.025 |
| H | 2.3864 | 7.1093 | 11.9799 |  | 2.414 | 7.3669 | 12.00721 |
| C | 2.6934 | 5.937 | 10.2502 |  | 2.6578 | 6.07058 | 10.19375 |
| C | 3.7515 | 5.0741 | 10.5622 |  | 3.77902 | 5.28703 | 10.53166 |
| C | 4.3064 | 4.0911 | 9.7347 |  | 4.39211 | 4.31532 | 9.7146 |
| C | 5.3447 | 3.1575 | 10.0749 |  | 5.51391 | 3.49192 | 10.08765 |
| H | 5.7629 | 3.0894 | 10.9021 |  | 6.03264 | 3.54728 | 11.03337 |
| C | 5.61 | 2.4039 | 8.9994 |  | 5.76941 | 2.64423 | 9.04847 |
| H | 6.2548 | 1.7362 | 8.9442 |  | 6.53431 | 1.88368 | 8.99406 |
| C | 4.7227 | 2.8104 | 7.9476 |  | 4.81126 | 2.92465 | 8.00946 |
| C | 4.6489 | 2.2308 | 6.6745 |  | 4.69904 | 2.23244 | 6.78574 |
| C | 4.3623 | 5.2011 | 11.9348 |  | 4.34009 | 5.46703 | 11.91123 |
| C | 5.5398 | 5.8452 | 12.1042 |  | 5.49244 | 6.233 | 12.13024 |
| H | 5.9625 | 6.231 | 11.3714 |  | 5.98783 | 6.71105 | 11.28962 |
| C | 6.1256 | 5.9345 | 13.3609 |  | 6.00936 | 6.39828 | 13.41728 |
| H | 6.9308 | 6.3881 | 13.4684 |  | 6.89951 | 6.99829 | 13.57146 |
| C | 5.5217 | 5.3617 | 14.4261 |  | 5.36488 | 5.78705 | 14.49187 |
| C | 4.3831 | 4.7103 | 14.2612 |  | 4.21941 | 5.01561 | 14.29841 |
| H | 3.9803 | 4.2963 | 14.9905 |  | 3.72457 | 4.53636 | 15.13593 |
| C | 3.7797 | 4.6349 | 13.0103 |  | 3.7101 | 4.85942 | 13.00752 |
| H | 2.9681 | 4.1912 | 12.9146 |  | 2.82368 | 4.25131 | 12.85 |
| C | 5.7328 | 1.2551 | 6.3254 |  | 5.65548 | 1.10024 | 6.55714 |
| C | 5.4729 | -0.088 | 6.058 |  | 5.23009 | -0.22428 | 6.73926 |
| H | 4.6009 | -0.4084 | 6.1051 |  | 4.20881 | -0.42343 | 7.05194 |
| C | 6.5081 | -0.9504 | 5.7238 |  | 6.1113 | -1.28957 | 6.5414 |
| H | 6.3334 | -1.8423 | 5.5289 |  | 5.77021 | -2.30803 | 6.69227 |
| C | 7.7991 | -0.465 | 5.6822 |  | 7.42532 | -1.02591 | 6.1556 |
| C | 8.0865 | 0.847 | 5.9644 |  | 7.86983 | 0.28194 | 5.96715 |
| H | 8.9627 | 1.1593 | 5.9389 |  | 8.89115 | 0.48323 | 5.66358 |
| C | 7.0348 | 1.7006 | 6.2868 |  | 6.98077 | 1.33936 | 6.17089 |
| H | 7.2157 | 2.5919 | 6.4829 |  | 7.32104 | 2.36038 | 6.02159 |
| F | 2.3716 | 8.508 | 6.4324 |  | 2.83789 | 8.61879 | 7.51489 |
| F | 2.1095 | 9.7289 | 4.0808 |  | 2.27799 | 10.59709 | 5.8073 |
| F | 4.4503 | 6.2121 | 2.0144 |  | 3.10972 | 7.69785 | 2.14726 |
| F | 4.6915 | 4.9872 | 4.3437 |  | 3.66821 | 5.70516 | 3.84097 |
| O | 3.6582 | 6.0785 | 6.6463 |  | 3.56444 | 6.0966 | 6.59034 |
| C | 3.5232 | 6.6916 | 5.4787 |  | 3.28578 | 7.08123 | 5.74871 |
| C | 2.8784 | 7.9204 | 5.3568 |  | 2.9389 | 8.37476 | 6.19085 |
| C | 2.7753 | 8.5406 | 4.1224 |  | 2.64595 | 9.40138 | 5.30159 |
| C | 3.2578 | 8.0274 | 2.9904 |  | 2.70548 | 9.21606 | 3.92552 |
| H | 3.1559 | 8.4553 | 2.1718 |  | 2.47187 | 10.01818 | 3.23692 |
| C | 3.9244 | 6.812 | 3.1092 |  | 3.06123 | 7.95151 | 3.47147 |
| C | 4.0378 | 6.1601 | 4.3036 |  | 3.35531 | 6.916 | 4.3499 |
| **5-F model** | | | | | | | |
| Sn | 6.7105 | 4.0979 | 13.5585 |  | 6.71047 | 4.0979 | 13.55848 |
| F | 10.2635 | 4.1127 | 15.2053 |  | 9.647 | 4.77405 | 16.11394 |
| F | 12.6811 | 3.916 | 14.0661 |  | 12.31783 | 4.55077 | 15.99403 |
| F | 13.2045 | 5.1601 | 11.7137 |  | 13.66276 | 5.04516 | 13.64685 |
| F | 11.2435 | 6.6296 | 10.5065 |  | 12.22737 | 5.7345 | 11.40603 |
| F | 8.8035 | 6.7738 | 11.5906 |  | 9.55666 | 5.9623 | 11.50651 |
| I | 0.8609 | 6.8696 | 20.9753 |  | 1.46269 | 6.97092 | 21.45704 |
| I | 11.342 | -2.51 | 19.1262 |  | 12.00638 | -2.04567 | 19.24787 |
| N | 5.3115 | 5.6133 | 13.8701 |  | 5.30545 | 5.60055 | 13.92401 |
| N | 6.6163 | 3.6381 | 15.6007 |  | 6.72017 | 3.61287 | 15.59731 |
| O | 8.2158 | 5.4699 | 13.9579 |  | 8.16362 | 5.50137 | 13.87373 |
| C | 4.7574 | 6.3862 | 12.8635 |  | 4.70948 | 6.39159 | 12.96338 |
| C | 3.6623 | 7.1074 | 13.4254 |  | 3.71709 | 7.21028 | 13.61191 |
| H | 3.1203 | 7.7102 | 12.9699 |  | 3.0863 | 7.9296 | 13.11067 |
| C | 3.5425 | 6.7706 | 14.7256 |  | 3.72029 | 6.89186 | 14.93951 |
| H | 2.8993 | 7.0858 | 15.3184 |  | 3.09192 | 7.30382 | 15.71522 |
| C | 4.5909 | 5.8346 | 15.028 |  | 4.71664 | 5.87079 | 15.14209 |
| C | 4.8331 | 5.2174 | 16.2697 |  | 5.00136 | 5.2311 | 16.36674 |
| C | 5.794 | 4.2323 | 16.5359 |  | 5.94405 | 4.20256 | 16.56728 |
| C | 6.0572 | 3.6053 | 17.7925 |  | 6.2554 | 3.582 | 17.83196 |
| H | 5.6521 | 3.8176 | 18.6023 |  | 5.80149 | 3.84286 | 18.77652 |
| C | 6.9903 | 2.6579 | 17.6038 |  | 7.20834 | 2.6342 | 17.60113 |
| H | 7.3375 | 2.0964 | 18.2585 |  | 7.67361 | 1.97859 | 18.32233 |
| C | 7.3547 | 2.6628 | 16.2309 |  | 7.5025 | 2.64904 | 16.18922 |
| C | 8.2917 | 1.817 | 15.605 |  | 8.43735 | 1.82122 | 15.53659 |
| C | 3.9383 | 5.6248 | 17.4021 |  | 4.18644 | 5.64447 | 17.55565 |
| C | 3.1269 | 4.67 | 18.0312 |  | 3.14101 | 4.82437 | 18.00621 |
| H | 3.1725 | 3.7807 | 17.7616 |  | 2.92117 | 3.89735 | 17.48417 |
| C | 2.2633 | 5.0281 | 19.0424 |  | 2.3672 | 5.19523 | 19.10772 |
| H | 1.7224 | 4.3875 | 19.445 |  | 1.55917 | 4.55325 | 19.4409 |
| C | 2.2061 | 6.3395 | 19.4532 |  | 2.64376 | 6.3944 | 19.76376 |
| C | 2.9977 | 7.2967 | 18.8504 |  | 3.67679 | 7.22613 | 19.33358 |
| H | 2.9532 | 8.1825 | 19.1301 |  | 3.89073 | 8.15712 | 19.8469 |
| C | 3.86 | 6.9336 | 17.8259 |  | 4.44248 | 6.84619 | 18.22908 |
| H | 4.3909 | 7.5804 | 17.4207 |  | 5.25166 | 7.48875 | 17.89311 |
| C | 8.9854 | 0.8188 | 16.4676 |  | 9.25886 | 0.91145 | 16.40161 |
| C | 8.7364 | -0.5344 | 16.2895 |  | 8.94909 | -0.44939 | 16.52218 |
| H | 8.12 | -0.8119 | 15.6508 |  | 8.09443 | -0.85544 | 15.9881 |
| C | 9.4054 | -1.4752 | 17.0609 |  | 9.72113 | -1.29144 | 17.32597 |
| H | 9.233 | -2.3821 | 16.9472 |  | 9.4662 | -2.34195 | 17.41275 |
| C | 10.3178 | -1.0564 | 17.9919 |  | 10.81442 | -0.76269 | 18.01079 |
| C | 10.5639 | 0.2664 | 18.2036 |  | 11.14481 | 0.5879 | 17.90323 |
| H | 11.1684 | 0.5313 | 18.8587 |  | 11.99961 | 0.99409 | 18.43255 |
| C | 9.9071 | 1.2122 | 17.4381 |  | 10.3639 | 1.42091 | 17.09955 |
| H | 10.0812 | 2.1157 | 17.5708 |  | 10.62238 | 2.47185 | 17.00468 |
| C | 9.4252 | 5.4158 | 13.4156 |  | 9.48344 | 5.38612 | 13.81766 |
| C | 10.4628 | 4.72 | 14.0222 |  | 10.25928 | 5.04358 | 14.94333 |
| C | 11.7134 | 4.6429 | 13.4411 |  | 11.6438 | 4.92262 | 14.89224 |
| C | 11.9757 | 5.2478 | 12.284 |  | 12.32868 | 5.16603 | 13.70475 |
| C | 10.9942 | 5.9985 | 11.6668 |  | 11.59782 | 5.52165 | 12.57429 |
| C | 9.7354 | 6.0641 | 12.2376 |  | 10.21342 | 5.63902 | 12.63871 |
| I | 12.5601 | 1.3262 | 6.1417 |  | 11.95834 | 1.22498 | 5.65995 |
| I | 2.0789 | 10.7058 | 7.9908 |  | 1.41468 | 10.24159 | 7.8691 |
| N | 8.1095 | 2.5825 | 13.2469 |  | 8.11549 | 2.59525 | 13.19295 |
| N | 6.8047 | 4.5577 | 11.5163 |  | 6.70077 | 4.58293 | 11.51965 |
| C | 8.6636 | 1.8096 | 14.2535 |  | 8.71147 | 1.80421 | 14.15358 |
| C | 9.7587 | 1.0884 | 13.6916 |  | 9.70386 | 0.98553 | 13.50505 |
| H | 10.3006 | 0.4856 | 14.1471 |  | 10.33466 | 0.26621 | 14.0063 |
| C | 9.8785 | 1.4252 | 12.3914 |  | 9.70066 | 1.30395 | 12.17745 |
| H | 10.5217 | 1.11 | 11.7986 |  | 10.32904 | 0.892 | 11.40174 |
| C | 8.8301 | 2.3612 | 12.089 |  | 8.70431 | 2.32502 | 11.97487 |
| C | 8.5878 | 2.9784 | 10.8473 |  | 8.41959 | 2.96471 | 10.75022 |
| C | 7.6269 | 3.9635 | 10.5811 |  | 7.4769 | 3.99324 | 10.54969 |
| C | 7.3638 | 4.5905 | 9.3245 |  | 7.16556 | 4.61382 | 9.285 |
| H | 7.7689 | 4.3782 | 8.5147 |  | 7.61947 | 4.35296 | 8.34044 |
| C | 6.4307 | 5.5379 | 9.5132 |  | 6.21262 | 5.56162 | 9.51583 |
| H | 6.0835 | 6.0994 | 8.8585 |  | 5.74736 | 6.21724 | 8.79464 |
| C | 6.0662 | 5.533 | 10.8861 |  | 5.91845 | 5.54677 | 10.92774 |
| C | 5.1293 | 6.3788 | 11.512 |  | 4.9836 | 6.37459 | 11.58037 |
| C | 9.4826 | 2.571 | 9.7149 |  | 9.23453 | 2.55136 | 9.56132 |
| C | 10.2941 | 3.5258 | 9.0858 |  | 10.27996 | 3.37147 | 9.11078 |
| H | 10.2484 | 4.4151 | 9.3554 |  | 10.49979 | 4.29849 | 9.63284 |
| C | 11.1576 | 3.1677 | 8.0746 |  | 11.05379 | 3.00063 | 8.00928 |
| H | 11.6986 | 3.8083 | 7.672 |  | 11.86182 | 3.64262 | 7.67612 |
| C | 11.2149 | 1.8563 | 7.6638 |  | 10.77725 | 1.80147 | 7.35322 |
| C | 10.4233 | 0.8991 | 8.2666 |  | 9.74421 | 0.96973 | 7.78337 |
| H | 10.4678 | 0.0133 | 7.9869 |  | 9.53028 | 0.03875 | 7.27003 |
| C | 9.561 | 1.2622 | 9.2911 |  | 8.9785 | 1.34965 | 8.88786 |
| H | 9.0301 | 0.6154 | 9.6963 |  | 8.16932 | 0.70709 | 9.22381 |
| C | 4.4355 | 7.377 | 10.6494 |  | 4.16211 | 7.28439 | 10.71536 |
| C | 4.6846 | 8.7302 | 10.8275 |  | 4.47191 | 8.64523 | 10.59481 |
| H | 5.301 | 9.0077 | 11.4662 |  | 5.32658 | 9.05125 | 11.1289 |
| C | 4.0156 | 9.671 | 10.0561 |  | 3.69989 | 9.4873 | 9.79102 |
| H | 4.1879 | 10.5779 | 10.1698 |  | 3.95484 | 10.5378 | 9.70425 |
| C | 3.1031 | 9.2522 | 9.1251 |  | 2.6066 | 8.95858 | 9.10618 |
| C | 2.857 | 7.9294 | 8.9134 |  | 2.27619 | 7.60799 | 9.21372 |
| H | 2.2526 | 7.6645 | 8.2583 |  | 1.42138 | 7.20182 | 8.68439 |
| C | 3.5139 | 6.9836 | 9.6789 |  | 3.05707 | 6.77495 | 10.0174 |
| H | 3.3398 | 6.0801 | 9.5462 |  | 2.79858 | 5.72401 | 10.11224 |
| F | 3.1575 | 4.0831 | 11.9117 |  | 3.77381 | 3.42181 | 11.00313 |
| F | 0.7399 | 4.2798 | 13.0509 |  | 1.10297 | 3.64499 | 11.12319 |
| F | 0.2164 | 3.0357 | 15.4033 |  | -0.24182 | 3.1504 | 13.4704 |
| F | 2.1775 | 1.5662 | 16.6105 |  | 1.1937 | 2.46097 | 15.71111 |
| F | 4.6174 | 1.422 | 15.5264 |  | 3.86441 | 2.23328 | 15.61048 |
| O | 5.2051 | 2.7259 | 13.1591 |  | 5.25732 | 2.69442 | 13.24322 |
| C | 3.9958 | 2.78 | 13.7014 |  | 3.9375 | 2.80962 | 13.29937 |
| C | 2.9582 | 3.4758 | 13.0948 |  | 3.16159 | 3.15219 | 12.17376 |
| C | 1.7076 | 3.5529 | 13.6759 |  | 1.77707 | 3.2731 | 12.22493 |
| C | 1.4453 | 2.948 | 14.833 |  | 1.09226 | 3.02959 | 13.41243 |
| C | 2.4267 | 2.1973 | 15.4502 |  | 1.82319 | 2.67393 | 14.54284 |
| C | 3.6856 | 2.1317 | 14.8794 |  | 3.20759 | 2.55661 | 14.47834 |

Atomic polarizabilities and dipolar electrostatic potentials were calculated as follows. Firstly, geometry minimization and electron density determination of the five-substituted porphyrin rings, ranging from one to five fluorine atoms in each phenyl group, were performed using Gaussian 16 program,^[58]^ at the B3LYP/6-31++G* + LANL2DZdp (for I and Sn atoms) level of theory. Partitioning was performed using AIMAll software,^[61]^ employing Bader’s Theory of Atoms in Molecules (QTAIM).^[62]^ QTAIM provides an accurate definition of atomic regions with a possibility to shift the origin of each atomic basin to bond critical points (BCPs), thus enabling good transferability of atomic properties, such as dipole moments and polarizabilities. Those electric moments were obtained employing PolaBer software, *via* differentiation of atomic dipole moments when applying an external electric field of 0.001au, in respect to its non-perturbed offspring. Multiwfn was used to calculate the ESP and its critical points, mapped over an isoelectronic density surface at 0.001au, utilizing the wavefunctions obtained for the calculation of polarizabilities.^[63]^

**References:**

52. A. D. Adler, F. R. Longo, J. D. Finarelli, J. Goldmacher, J. Assour, L. Korsakoff *J. Org. Chem.* **1967**, 32, 476.

53. R. Patra, H. M. Titi, I. Goldberg *Cryst. Growth Des*. **2013**, 13, 1342.

54. Sheldrick GM. SHELXS version-**2018**/3 and SHELXL version-**2018**/3: programs for crystal structure solution and refinement. University of Gottingen, Germany, **2018**.

55. Sheldrick, G. M. SHELXS Version-2018/3 and SHELXL Version-2018/3: Programs for Crystal Structure Solution and Refinement; University of Gottingen: Germany, 2018.

56. Farrugia, L. *J. J. Appl. Cystallogr.* 1999, 32, 837−838.

57. P.R. Spackman, M. J. Turner, J.J. McKinnon, S.K. Wolff, D.J. Grimwood, D. Jayatilaka, M.A. Spackman *J. Appl. Cryst.* **2021** 54, 3, 1006–1011

58. M. J. Frisch, G. W. Trucks, H. B. Schlegel, G. E. Scuseria, M. A. Robb, J. R. Cheeseman, G. Scalmani, V. Barone, G. A. Petersson, H. Nakatsuji, X. Li, M. Caricato, A. V. Marenich, J. Bloino, B. G. Janesko, R. Gomperts, B. Mennucci, H. P. Hratchian, J. V. Ortiz, A. F. Izmaylov, J. L. Sonnenberg, D. Williams-Young, F. Ding, F. Lipparini, F. Egidi, J. Goings, B. Peng, A. Petrone, T. Henderson, D. Ranasinghe, V. G. Zakrzewski, J. Gao, N. Rega, G. Zheng, W. Liang, M. Hada, M. Ehara, K. Toyota, R. Fukuda, J. Hasegawa, M. Ishida, T. Nakajima, Y. Honda, O. Kitao, H. Nakai, T. Vreven, K. Throssell, J. A. Montgomery, Jr., J. E. Peralta, F. Ogliaro, M. J. Bearpark, J. J. Heyd, E. N. Brothers, K. N. Kudin, V. N. Staroverov, T. A. Keith, R. Kobayashi, J. Normand, K. Raghavachari, A. P. Rendell, J. C. Burant, S. S. Iyengar, J. Tomasi, M. Cossi, J. M. Millam, M. Klene, C. Adamo, R. Cammi, J. W. Ochterski, R. L. Martin, K. Morokuma, O. Farkas, J. B. Foresman and D. J. Fox, Gaussian 16, Revision C.01, Gaussian, Inc., Wallingford CT, 2016.

59. Becke, A. D. *J. Chem. Phys*. **1993,** *98*, 5648

60. Lee, C.; Yang, W.; Parr, R. G. *Phys. Rev. B* **1988**, *37*, 785.

61. T. A. Keith, AIMAll (Version 19.10.12), TK Gristmill Software, Overland Park KS, USA, 2019, <http://aim.tkgristmill.com>.

62. R.F.W. Bader, Atoms in Molecules, a Quantum Theory, Oxford University Press, 1990.

63. T. Lu and F. Chen, *J. Comput. Chem*., 2012, 33, 580-592.
